# Amyloid, tau, pathogen infection and antimicrobial protection in Alzheimer’s disease –conformist, nonconformist, and realistic prospects for AD pathogenesis

**DOI:** 10.1186/s40035-018-0139-3

**Published:** 2018-12-24

**Authors:** Hongmei Li, Chia-Chen Liu, Hui Zheng, Timothy Y. Huang

**Affiliations:** 1 Department of Neuroscience, Mayo Clinic, Jacksonville, FL USA; 2 Huffington Center on Aging, Baylor College of Medicine, Houston, TX USA; 3 Neuroscience Initiative, Sanford Burnham Prebys Medical Discovery Institute, San Diego, CA USA

**Keywords:** Amyloid-beta, Alzheimer’s disease, Amyloid hypothesis, Tau hypothesis, Pathogen hypothesis, Viral infection, Antimicrobial protection

## Abstract

**Background:**

Alzheimer’s disease (AD) is a fatal disease that threatens the quality of life of an aging population at a global scale. Various hypotheses on the etiology of AD have been developed over the years to guide efforts in search of therapeutic strategies.

**Main body:**

In this review, we focus on four AD hypotheses currently relevant to AD onset: the prevailing amyloid cascade hypothesis, the well-recognized tau hypothesis, the increasingly popular pathogen (viral infection) hypothesis, and the infection-related antimicrobial protection hypothesis. In briefly reviewing the main evidence supporting each hypothesis and discussing the questions that need to be addressed, we hope to gain a better understanding of the complicated multi-layered interactions in potential causal and/or risk factors in AD pathogenesis. As a defining feature of AD, the existence of amyloid deposits is likely fundamental to AD onset but is insufficient to wholly reproduce many complexities of the disorder. A similar belief is currently also applied to hyperphosphorylated tau aggregates within neurons, where tau has been postulated to drive neurodegeneration in the presence of pre-existing Aβ plaques in the brain. Although infection of the central nerve system by pathogens such as viruses may increase AD risk, it is yet to be determined whether this phenomenon is applicable to all cases of sporadic AD and whether it is a primary trigger for AD onset. Lastly, the antimicrobial protection hypothesis provides insight into a potential physiological role for Aβ peptides, but how Aβ/microbial interactions affect AD pathogenesis during aging awaits further validation. Nevertheless, this hypothesis cautions potential adverse effects in Aβ-targeting therapies by hindering potential roles for Aβ in anti-viral protection.

**Conclusion:**

AD is a multi-factor complex disorder, which likely requires a combinatorial therapeutic approach to successfully slow or reduce symptomatic memory decline. A better understanding of how various causal and/or risk factors affecting disease onset and progression will enhance the likelihood of conceiving effective treatment paradigms, which may involve personalized treatment strategies for individual patients at varying stages of disease progression.

## Background

Alzheimer’s disease (AD) is the number one cause of age-related dementia, currently with no effective therapy. It represents an imminent threat to the health-span of the senior population. With unprecedented growth of a globally aging population, AD will become an increasing burden to society if left unchecked [1–6]. The clinical manifestations of AD appear with incidental forgetfulness at initial stages, eventually progressing to mild cognitive impairment to full-blown AD with noticeable difficulties in cognitive functions such as memory, planning and organizing. Patients at later stages of AD not only suffer severe decline in cognitive functions, but also may experience drastic personality and behavioral changes, with an eventual incapacity to independently carry out daily functions [7–10]. Although these clinical symptoms may be indicative of AD onset, definitive diagnosis requires the detection of three pathological hallmarks in the brain, namely, extracellular amyloid (plaques) composed of Aβ peptides (graded by Thal phases [11, 12]), intracellular neurofibrillary tangles (NFTs) formed by hyperphosphorylated tau (categorized through Braak staging [13–15]), and degeneration in brain regions such as the entorhinal cortex, hippocampus, and cerebral cortex during late stages of onset [7, 16]. Previously, these pathological features associated with AD could only be confirmed by postmortem analysis. With rapidly evolving developments in the brain imaging field, it is currently possible to observe amyloid and tau aggregates and degeneration within the central nervous system (CNS) during ante-mortem stages [17–22]. Given the current advances in neuroimaging, together with the identification of diagnostic biomarkers in cerebrospinal fluid (CSF) and/or blood [23–31], the possibility of diagnosing and monitoring AD from early onset to terminal stages of AD progression, and characterizing the efficacy of various therapeutic treatments has become more promising.

It has been debated whether pathological hallmarks, such as plaques and NFTs have an active role in driving disease progression, or whether they merely reflect progression and severity of the disease. Postmortem studies and neuroimaging results indicate the appearance of pathological features associated with AD in the brain decades before the onset of cognitive symptoms [7, 19, 32]. Should the pathologies function as drivers of the disease, removal or reduction of these pathological features will naturally be beneficial in preventing, slowing down or even reversing the disease progression. In the case where the pathological hallmarks are merely byproducts of disease onset, then targeting these markers would have little or no beneficial effect, necessitating the search for the primary trigger for AD onset.

Multiple hypotheses have evolved regarding the primary initiator of AD onset based on clinical observations, which is supported by results derived from various experimental model systems. In this review, we will briefly discuss four influential hypotheses for AD: the amyloid cascade hypothesis that has dominated the field for decades, the tau hypothesis that gained much more attention following repeated clinical failures using Aβ-centered targeting strategies, the pathogen hypothesis that has gained supporting evidence from recent publications, and the “antimicrobial protection hypothesis” that revisits the amyloid hypothesis in the context of microbial pathogen response. Although other important AD-related hypotheses exist which involve mitochondrial/oxidative stress, insulin-resistance, cerebrovascular dysfunction, and neuroinflammation, etc., we defer our discussion here to many excellent reviews that comprehensively discuss these topics (e.g. [33–49]).

Due to the vast literature related to the topics covered in this review, we are limited to the number of studies cited in our discussion below; thus, we apologize to the authors whose contributions have advanced the field, but whose work are not referenced herein.

## Main text

### Amyloid cascade hypothesis

The amyloid cascade hypothesis (referred to as the “amyloid hypothesis” hereon) has undoubtedly had the greatest influence on AD research for nearly three decades. The amyloid hypothesis originally proposed a role for amyloid plaques as a causal initiator for all downstream pathological events in AD onset, including NFTs and neurodegeneration in the CNS [50]. The hypothesis has been modified since to propose a primary role for soluble Aβ oligomers as the critical driver in AD progression [51–56]. The amyloid hypothesis lends strong support from genetic evidence in familial AD (fAD) comprising a small percentage of hereditary AD cases and countless experimental model systems. However, the key question remains whether familial AD is equivalent to sporadic AD (sAD), representing over 95% of all AD cases. Several recent reviews have summarized arguments supporting and contradicting the amyloid hypothesis [57–60].

Amyloid plaques are pathological aggregates comprising amyloid-β (Aβ) peptides, derived from sequential proteolysis of amyloid precursor protein (APP) by β- and γ-secretases. Autosomal dominant mutations all reside in three genes, APP, *PSEN1* and *PSEN2*, with the latter two encoding the catalytic subunit of the γ-secretase complex [2, 61–63]. These mutations are invariably linked to increased generation/accumulation of the aggregation-prone Aβ42 peptide. While sporadic AD manifests usually at the age of seventy to eighty or older, fAD mutations may trigger early AD onset (before the age of 65 years old) [64], and sometimes can be as early as age 30 in the mutation carriers (https://www.alzheimers.net/10-13-14-early-onset-alzheimers/). Although fAD patients represent less than 5% of the AD population, their pathological and clinical characteristics provide critical genetic evidence that increased Aβ42 levels in the CNS can almost invariably aggravate AD onset. Aβ accumulation in sporadic AD has been thought to be primarily derived from reduced Aβ clearance rather than enhanced Aβ generation from clinical analyses in human CSF [65]. In support of the notion that sAD is associated with impaired Aβ efflux in the CNS, expression of human apolipoprotein E4 (apoE4), the most potent risk factor for sAD characterized to date, is seen to potently reduce Aβ clearance and increases Aβ plaque load [37, 66–68]. Thus, an imbalance between Aβ production and clearance potentially leads to Aβ accumulation in both familial and sporadic cases of AD, where different mechanisms drive elevations in Aβ levels in the CNS.

With respect to how Aβ accumulation can trigger AD onset, thousands of publications have proposed various mechanisms describing how Aβ can mediate neuronal dysfunction in vitro and in vivo. A non-exhaustive list of toxic effects associated with Aβ include impairments in synaptic plasticity and synaptic loss as observed in human AD brain [54–56, 69–72], dysregulation of calcium homeostasis which occurs prior to synaptic impairment [73–80], dysfunctional axonal trafficking [81–85], functional perturbation of cellular organelles such as lysosomes, endoplasmic reticulum, Golgi and mitochondria [86–95], and induction of astrogliosis and neuroinflammation [96–103]. Although most mouse models expressing human β-amyloid fail to induce NFTs comprising mouse tau within the murine lifespan [104–106], the presence of Aβ oligomers or APP with fAD mutation(s) is invariably associated with increased tau pathology in tau transgenic mice [107, 108], tauopathy models in rats [109], non-human primates [110], and in 3D human iPSC (induced pluripotent stem cell)-derived neuronal cell culture systems [111–114]. Moreover, neurodegeneration has been observed in human iPSC-derived neurons grafted into the brain of 5xFAD transgenic mice [115], suggesting that human neurons may be more susceptible to Aβ toxicity compared to mouse neurons. In short, regardless of whether Aβ accumulation is caused by amyloidogenic APP processing or reduced Aβ clearance, a vast sum of literature supports the role of Aβ elevation in triggering deleterious events associated with neurodegeneration.

Despite this, the amyloid hypothesis is also unable to satisfy many observations that counter the significance or potency of Aβ in sAD [58, 60, 116–118]. Inconsistencies with respect to a primary role for Aβ in triggering AD onset include: [1] poor correlation between amyloid plaque load and severity of cognitive impairment/degeneration in human brain; further, many individuals feature an abundant amyloid plaque load in the brain without manifesting deficits in cognitive function [119–126], [2] most human β-amyloid mouse models are not associated with NFT pathology as mentioned above, and [3] many Aβ-centered clinical trials show little or no efficacy (as reviewed in [116, 127, 128]). Alternative explanations may preclude outright rejection of the amyloid hypothesis [57, 58, 129, 130]; it may be more favorable to modify or elaborate on the amyloid hypothesis to accommodate its inability to comprehensively predict the outcomes mentioned above. For example, a potential delay in Aβ-mediated proteotoxicity due to intrinsic neuroprotective mechanisms within the brain would account for cognitively non-impaired individuals with elevated amyloid loads. A pathogenic synergy may be apparent between Aβ and tau, which would also explain a poor correlation between amyloid load and cognition: the formation of neuritic plaques which feature amyloid filaments that coincide with swollen/dystrophic neurites correlate with early cognitive impairment more accurately than amyloid or tau pathology alone [131, 132]. Animal models may fail to fully recapitulate human AD pathology due to the cross-species differences in tau, and/or the short life-span of rodents in comparison with human beings. Failures in Aβ-targeted clinical trials may result from the disease stages of the participants upon treatment, or specificity of the Aβ species targeted by certain drugs in the trial, in addition to many other factors (e.g. drug potency). With promising results from a Phase II clinical trial characterizing protective effects of an anti-Aβ protofibril antibody (BAN2401), targeting the toxic forms of Aβ may yet be protective: News release from Esai and Biogen indicates a dose-dependent reduction in plaque load and reduced clinical decline in drug-treatment groups compared to placebos in an 856 mild cognitive impairment (MCI) patient cohort (http://investors.biogen.com/news-releases/news-release-details/eisai-and-biogen-announce-detailed-results-phase-ii-clinical).

In summary, it is likely that Aβ and amyloid plaques are necessary but may not be sufficient to initiate all the downstream events required for AD pathogenesis, especially in sporadic AD. However, Aβ pathology is unlikely to be an inconsequential epiphenomenon in AD pathogenesis, despite instances where seniors accumulate plaques in the brain for decades without showing overt cognitive deficits. In sporadic AD, dysregulated Aβ homeostasis may initiate late in life and gradually precondition the brain to be more susceptible to other internal and/or external insults during aging, while in fAD, chronic imbalance in Aβ levels derived from inherited amyloidogenic dominant mutations may erode endogenous defense mechanism and manifest AD pathology to an early AD onset. Despite certain inadequacies with the amyloid hypothesis, strategies underlying therapeutic AD drug design will need to consider newly discovered physiological roles for Aβ in microbial infection and pathogen response as reviewed in the discussion of “the antimicrobial protection hypothesis” below.

### Tau hypothesis

Given that Aβ pathology correlates poorly with cognitive decline, a central role for tau in driving AD onset has also been considered. The tau hypothesis proposes that tau is a fundamental pathogenic initiator that triggers all the downstream pathological events during AD onset. Unlike amyloid accumulation, pathological Braak stages - characterized tau tangles primarily comprising hyperphosphorylated tau, correlates more tightly with cognitive impairment and AD severity [13, 14, 120, 133–135]. The progressive onset of tau pathology and distinctive spatial propagation of tau tangles characterized using conventional postmortem pathological analysis and recent methods in tau PET imaging, implicates tau as a better prognostic indicator for neurodegeneration and cognitive deficits in AD compared to amyloid pathology [13, 14, 136–141]. Contrary to frequently observed instances of high brain amyloid with no apparent cognitive impairment, the occurrence of non-symptomatic individuates with advanced Braak pathology (Braak stages V-VI) is comparatively rare [142, 143]. Moreover, van Rossum et al reported that CSF total-tau and phosphorylated-tau (which are markers of CNS injury) are associated with rapid progression from MCI to late-stage dementia [144, 145]. These observations indicate that tauopathy can be a key factor driving AD onset and progression. This notion is supported by studies demonstrating a critical role for tau in mediating toxic effects derived from Aβ using tau deletion models in vitro and in vivo [146–153]. Should tau be an essential driver in AD onset, therapeutically targeting tau may effectively attenuate disease onset and AD pathogenesis.

Tau is a soluble microtubule-binding protein localized primarily to axons in adult neurons under normal physiological conditions. Tau directly binds microtubules, thereby promoting its assembly and stability. Six tau isoforms are derived from splice variation, where tau is also subject to regulation through various posttranslational modifiers (reviewed in [154–159]). Besides its predominant localization to axons, tau has also been detected at lower levels in dendrites, and at the plasma membrane, Golgi complex, rough endoplasmic reticulum, nucleus and nucleolus, (reviewed [159–161]). Tau function extends beyond microtubule-binding, and includes roles in regulating synaptic function [161], protecting RNA and DNA in early stress response [162, 163], and affecting nucleocytoplasmic transport via direct interaction with nucleoporins [164]. A majority of studies so far have focused on tau hyperphosphorylation, since pathological tau phosphoforms are prone to aggregation and feature various deficiencies in physiological functions, in addition to driving dominant effects in toxicity [165–167]. In addition to hyperphosphorylation, tau acetylation and glycosylation can also aggravate AD-associated tau pathology, whereas tau O-GlcNAc modification is likely protective and is seen to attenuate tau pathology in several transgenic models [168–175]. Additional posttranslational modifications such as truncation [176–179], ubiquitylation [180, 181], SUMOylation [182], and nitration [183–185] have been reported for tau, with aberrant modifications found to be associated with either hyperphosphorylation, increased tau aggregation or mis-localization of tau in vivo or in vitro (recently reviewed [159, 186, 187]).

Pathological tau dysfunction is primarily thought to be derived from a loss in microtubule binding, thereby leading to many downstream events in cellular dysregulation such as impairment in mitochondrial transport and functions [188–192], synaptic deficits [170, 193–196], defective axonal transportation [197–200], and enhanced stress granule formation [201–208]. Tau dysfunction can also alter neurogenesis in various mouse models; a transgenic line expressing human tau features reduced adult neurogenesis prior to the formation of pathological tau aggregates [209]. Conversely, models expressing human tau constructs that are refractory to aggregation exhibit increased neurogenesis [210]. Interestingly, tau deletion confers resistance to impairments in neurogenesis induced by chronic stress compared to wild type littermates, implicating a role for tau in suppressing neurogenesis [211]. A role for tau in AD-associated neuroinflammation has also been proposed, where inflammation can either be protective or deleterious to CNS function depending on the duration and extent of the inflammatory response (e.g. recently reviewed in [212–215]). In addition to driving neuroinflammation, the formation and distribution of pathological tau aggregates may be consequently modulated through pro-inflammatory triggers [216–219]. Although tau aggregates have been proposed to spread through prion-like mechanisms [220], these models fail to explain certain spatiotemporal aspects of NFTs in human postmortem brain [221]. How tau aggregates propagate in the brain is still actively under investigation. As an intracellular protein component, tau spreading requires extrusion and uptake from the extracellular environment via exome-dependent or independent mechanisms as indicated by various assays in vitro and in vivo [222–226]. AD is also associated with insulin resistance in the brain, which can both manifest in or occur independently of diabetes [227–230]; tau protein and insulin signaling pathways in the brain can also interact to exacerbate disease onset in aging and AD [231–233]. Future study will further define pathways and mechanisms by which tau can potentially modulate AD onset.

Although mounting evidence indicates an indispensable role for tau in AD, the tau hypothesis is still subject to criticism. Importantly, while tau mutations have been found to be associated with human tauopathy disorders such as frontotemporal lobar degeneration with tau inclusions (FTLD-tau), Pick’s disease, progressive supranuclear palsy (PSP), corticobasal degeneration (CBD), argyrophilic grain disease (AGD), and chronic traumatic encephalopathy, without amyloid accumulation [154, 234–237], these mutations are not linked to AD. This suggests that tau pathology may be induced as a downstream consequence of AD onset. However, hyperphosphorylated tau can be detected in the absence of tangles in young human brain as early as 14 years of age [14, 238, 239], where intraneuronal tau aggregates can be found across a large age group, indicating that tau pathogenesis is not necessarily dependent on age and AD-related toxicity. This also suggests that tau alone may not be a sufficient driver for AD onset. To complicate matters, the protective APOE variant in AD, APOE ε2, is associated with increased risk for tauopathy disorders such as PSP, CBD, and AGD [240–242]. Should tau be an upstream trigger for AD onset, it will be difficult to reconcile how protective APOE variants (APOE2) can be a risk factor for tauopathy. Several biomarker studies using human CSF demonstrate that ratios comprising Aβ42/total-tau and Aβ42/phospho-tau may accurately indicate transition from normal to mild cognitive impairment, and full AD onset [243–245], reiterating the importance of both markers in AD diagnosis. It is still currently unclear how Aβ and tau interact, and how these interactions lead to terminal cognitive phenotypes associated with AD. In the face of failures from amyloid-focused clinical trials, tau-targeting strategies have recently received growing attention. Although selective tau-aggregation blockers [246] or tau-reactive antibodies (https://clinicaltrials.gov/ct2/show/NCT00818662) have yet to succeed in meeting primary endpoints, additional efforts to develop active or passive vaccines and alternative strategies to modulate tau post-translational modification are being pursued [247] (recently reviewed in [59, 248]). Continued efforts are also currently being pursued with tau as an immune target; additional anti-tau antibodies are currently being developed and tested in preclinical stages (e. g [249–254]).

Similar to the role for Aβ in the context of the amyloid hypothesis, tau appears to also be necessary but insufficient as a primary initiator for AD. This is complicated by the observation that cognitively normal individuals can manifest brain amyloid or tau pathologies, or a combination of both [255, 256], which necessitates the identification of other mediators and modulators of AD.

### The “pathogen hypothesis” and “Antimicrobial Protection Hypothesis”

The “pathogen hypothesis”, or “infection hypothesis” in AD, suggests that chronic infection by viral, bacterial, and/or fungal pathogens may be a trigger for sporadic AD onset during aging. Candidate pathogens have been proposed in the literature over the years, including oral herpes - herpes simplex virus type 1 (HSV-1), genital herpes HSV-2, human herpesvirus 6A (HHV-6A) and HHV-7, Epstein Barr virus (EBV), cytomegalovirus, human immunodeficiency virus (HIV), gut bacteria, liver bacteria *Helicobacter pylori*, periodontal pathogens (bacteria linked to gingivitis), bacteria *Chlamydophila pneumoniae* that causes pneumonia, and others (recently reviewed in [257–261]). These pathogens may invade the CNS directly by translocating across the blood-brain-barrier and/or the brain-CSF barrier, through the trigeminal nervous system and oral-nasal pathway, or by penetrating the gastrointestinal tract [258, 262–267]. Moreover, pathogens may also secrete toxins circulating to the brain to dysregulate neurological functions associated with AD [260, 261, 268–277].

This concept that AD may be derived from infection was initially postulated by Dr. Oskar Fischer, who was regarded as Dr. Alzheimer’s rival when he independently reported the observation of pathological hallmarks associated with 12 dementia cases in 1907 [278–280]. Evidence in support of the pathogen hypothesis first appeared in 1991 from a group led by Dr. Ruth Itzhaiki, who has become one of the strongest advocates of the pathogen hypothesis in recent decades. This hypothesis was not well-accepted in the AD field until recent multi-omic studies surveying large populations have provided additional support to the infection hypothesis [281, 282].

The pathogen hypothesis has gained support from a recent study using large datasets across multiple independent clinical cohorts [282]. Combining multi-omic analyses on genomic, transcriptomic, and proteomic datasets, the study observed frequent viral infection in normal human brains. Interestingly, viral DNA and RNA from certain viral strains, namely human herpesvirus 6 (HHV-6) and herpesvirus 7 (HHV-7), were more abundant in AD samples, where viral DNA and RNA abundance were found to correlate with aggravated AD pathology [282]. AD risk has also been reported in association with chronic periodontitis in a ten-year retrospective, population-based study from Taiwan in 9291 patients diagnosed with chronic periodontitis in comparison with 18,672 non-infected patient controls [283]. Another recent study tracking HSV infection in 8362 infected individuals vs 25,086 sex- and age-matched controls in a Taiwan population observed that HSV infection significantly correlated with a higher risk of dementia later in life, where anti-herpetic treatment can greatly reduce risk of dementia onset [281]. Although no definitive conclusions can be made with respect to a causal role for HSV infection in AD, since APOE status and clinical AD characterization is lacking in the study, dramatic reduction in dementia risk with anti-herpetic treatment suggests that viral infection may be a serious risk factor that increases likelihood of dementia onset if left untreated. These recently discoveries have drawn increased attention to the pathogen hypothesis in AD which has been viewed skeptically for past two decades.

Although these recent findings do not sufficiently provide evidence as to whether viral infection is definitively causal to AD onset, these results provide compelling indication that viral infection may be a prevalent risk factor in dementia, and implicate a potential role for anti-viral strategies in AD therapy. The anti-viral agent valacyclovir (Valtrex) has been approved for testing in clinical trials by the FDA earlier this year for patients testing positive for herpes simplex virus-1 (HSV-1) or HSV-2 with mild dementia potentially due to AD (https://clinicaltrials.gov/ct2/show/NCT03282916).

Skepticism towards the pathogen hypothesis in the AD field likely stems from early published studies that derived data from small cohorts and sample sizes, and contradicting results from different groups; in addition, some studies fail to account for APOE status in AD and control groups. For example, the first study characterizing the presence of HSV-1 DNA in human postmortem brain samples was based on postmortem tissues from 8 AD patients and 6 normal controls, where HSV-1 DNA was detected in both AD and control samples [284]. Although, Dr. Itzhaiki’s group [284–290] together with others [291–296] have since confirmed the presence of viral DNA in human brain from different cohorts, conflicting results have been reported as to whether viral infection positively correlates with AD. For instance, while results from Dr. Itzhaiki’s group indicates a positive correlation between viral infection and AD onset, other reports failed to determine any correlation between viruses and AD [295–297]. HSV1 viral DNA is also found to be prevalent in human trigeminal ganglia, a sensory ganglion extending into the human brain; HSV1 infection has been reported across all age groups (from 0~10-year old up to over 90 age groups) and appears to be independent of gender [298]. Since studies have shown that women are more prone to AD and given that HSV1 infection appears to be gender independent, this may suggest that viral infection may not be a primary trigger for AD onset.

The validity of the pathogen hypothesis is further confounded by differential effects of APOE ε2, ε3, and ε4 alleles encoding apolipoprotein E2, E3, and E4 (apoE2, E3 and E4) variants. Although mechanisms for APOE-dependent AD onset remain under active investigation, it has been well established so far that APOE ε4 is the strongest risk factor for sAD, while ε2 appears to be protective in AD onset (recently reviewed in [37, 299]). Exactly how different apoE isoforms may affect viral infection in the body and CNS remains unclear, which makes it difficult to delineate contributions from viral infection to AD vs those from different apoE isoforms. For example, results indicating a positive correlation between HSV1 infection and AD risk comprises an imbalance in APOE ε4 carriers in AD and controls; where the percentage of APOE ε4 alleles was found to be > 10 times higher than controls in a study from 46 AD patients and 77 non-AD donors [300]. With respect to whether viral DNA correlates with amyloid plaque formation in AD [290], 5 out 6 AD patients were APOE ε4 carriers, where there was none APOE ε4 carrier in the control cases (the control group was made up of one APOE ε2 carrier and four APOE ε3/ε3 individuals). Although results from these studies indicate a positive association between HSV1 and AD, these results remain correlative and fail to establish viral infection as a primary trigger for AD onset. For instance, viral infection could possibly be a consequent event rather than instigator of AD onset; given that HSV1 DNA is localized to amyloid plaques, the proportion of HSV1 DNA in amyloid plaques in control patients was significantly reduced compared to AD brain as a consequence of lower plaque loads [290]. Alternatively, APOE alleles may modulate viral entry into the brain, whereby viral infection would be a consequence of APOE status rather than a causal factor for AD onset. Given these limitations, it is difficult to conclude that viral infection as an independent risk factor for AD, let alone causing AD.

In addition to the potential association of HSV1, HHV-6, and/or HHV-7 with AD, the gastrointestinal microbiome has also been proposed to play a role in AD pathogenesis by affecting the brain-gut-liver axis. Vogt et al. describe decreased Firmicutes and *Bifidobacterium* and increased Bacteroidetes in fecal samples from AD patients in comparison with controls (*n* = 25/group) [301]. Xu and Wang have identified AD-associated metabolites using an integrated computational approach utilizing publicly available databases including the human metabolome database [302]. MahmoudianDehkordi recently reported reduction of primary bile acid (cholic acid) and elevated bacterial-derived secondary bile acid (deoxycholic acid) in serum samples from AD patients [303]. Dysbiosis of microbiota and viruses can produce toxic metabolites that may breach the gut epithelial barrier, affect brain function, contribute to local and systemic inflammation, and cause dysregulation of tryptophan metabolism in the intestine that affect the production of various neurotransmitters including acetylcholine, gamma-aminobutyric acid (GABA), and serotonin (reviewed in [258, 304, 305]). Using a mouse amyloid model, Minter et al have reduced amyloid plaque load in APP_SWE_/PS1_ΔE9_ transgenic mouse brain by combinatorial antibiotic treatment [306, 307]. Harach et al observed that germ-free APP transgenic mice exhibited drastically reduced plaque pathology [308]. These observational and in vivo experimental studies support the notion that microbiota (bacteria, viruses, and bacteriophages etc) inhabiting within human body can contribute to AD pathology.

Many other lingering questions remain with respect to the pathogen hypothesis, and we refer readers to several excellent recent reviews that explore various aspects of this hypothesis [259, 260, 277, 309].

In parallel with recent progress in support of the pathogen hypothesis, a protective physiological role for Aβ as an antiviral peptide has been discovered [310–314]. It was initially proposed by Soscia et al when they reported Aβ40 and Aβ42 peptides inhibit the overnight growth of 8 out of a total of 12 types of bacteria and fungi tested [310]. Later, two groups found that Aβ could also inhibit replication of H3N2 and H1N1 influenza A virus *in vitro* [313], HSV-1 replication in fibroblast, epithelial, and neuronal cell lines [314]. Initial evidence in vivo indicate that 5xFAD mice and Aβ-expressing transgenic nematodes (*Caenorhabditis elegans*) survive longer than the control groups after exposure to gut pathogens (yeast *Candida albicans* or bacteria *Salmonella enterica* serovar Typhimurium) [311]. APP-knockout mice, on the other hand, have shortened survival after pathogenic challenge [311]. A recent publication from Drs. Tanzi and Moir’s group provided further evidence to support a protective antimicrobial role for Aβ. Using 3D human neural cell cultures and 5xFAD mice, these researchers demonstrated that HSV-1, HHV-6A, or HHV-6B infection accelerated β-amyloid deposition, where Aβ inhibits HSV-1 infection in the host cells and prolongs host survival after HSV-1-induced encephalitis in 5xFAD mice [312]. The antimicrobial effect of Aβ is mediated via its heparin-binding domain. These in vitro and in vivo results strongly support the hypothesis that Aβ peptides play an important role in brain’s innate immune system by entrapping invading pathogens, thereby protecting the brain from infection. The “antimicrobial protection hypothesis” in AD has been discussed extensively in a recent review article [315], detailing antimicrobial abilities of Aβ and drawing comparisons of Aβ with other well-known antimicrobial peptides (AMPs) [315]. Given that dysregulation of the other known AMPs can lead to host cell toxicity, AD may be triggered by chronic activation of sustained inflammation due to elevations in Aβ in response to an elevated microbial burden during aging.

In summary, evidence so far in support of the infection hypothesis indicates that the presence of viruses may be a risk for AD; however, due to the prevalence of pathogen existence within the human body throughout life, definitive evidence is lacking that infection is causal to AD. Similar to the amyloid hypothesis, the antimicrobial hypothesis indicates that Aβ accumulation is a key to AD pathogenesis. It is likely that sophisticated protective mechanisms have evolved for human brains to confer resilience to stress and dysfunction for decades in life. So far, multi-omics human data suggest HHV-6A and HHV-7 to be prominently associated with AD across 3 independent cohorts [282]. Defining the causal or consequential role of pathogen infection remains a challenge. The discovery of Aβ-mediated antimicrobial activity and pathogen-dependent effects in inducing Aβ aggregation have connected these two factors together with neuroinflammation, which represents a double-edged sword in AD pathogenesis. Human stem cell- or iPSC-derived 3D culture or organoid culture systems are possible experimental systems to study a role for pathogens in AD, in which exposures of different infective agents can be clearly managed. Studying effects of pathogen in animal models may require sterile animal house facilities to control the effects from specific bacteria, viruses, or fungi in AD pathogenesis.

### Conclusions & Future Prospective

Recent advances of the pathogen and Aβ-antimicrobial hypotheses have shifted an ever-evolving view of the etiological origin of AD. It provides novel insights for future therapeutic strategies in combining anti-amyloid strategies with anti-viral approaches to be tested in clinical trials. New discoveries published recently indicate that APP mRNA in the brain can be reverse-transcribed into DNA and reinserted into the genome, resulting in thousands of APP variants with point mutations, insertions, deletions and so on. This phenomenon is much more pronounced in brains of sporadic AD than those of non-AD controls [316]. These findings pose additional questions regarding whether viral infection has any contribution to the substantially high amount of APP variants in sAD brain. Authors of the paper also suggest that FDA-approved combined anti-retroviral therapy for HIV infection may be potentially effective in treating sAD, Down syndrome and fAD patients.

Many unanswered questions remain for AD pathogenesis; for example, do different isoforms of apoE and/or different apoE aggregation status (monomer, oligomer, and/or lipidated particles) play any role in modulating antimicrobial Aβ activity? What are the physiological events or mechanisms that link Aβ and tau pathology in AD? In addition to Aβ, do tau oligomers, α-synuclein oligomers also confer any antimicrobial effects in infected cell types in the CNS? Should the infection model/antimicrobial model be valid, will chronic viral infection in the amyloid mouse models lead to tau pathology and neurodegeneration during aging, representing an improved model system for AD?

Unlike familial AD, sporadic AD may evolve from a combination of various genetic and environmental factors. Neuroinflammation, tau pathogenesis, and viral infection have all been implicated to play important roles in AD; however, these factors do not appear to be pathogenic triggers that are specifically relevant to AD. Thus, specific causal mechanisms that drive AD onset have yet to be clearly defined, which may lead to the identification of new therapeutic targets. It is now widely accepted that sporadic AD is a complicated syndrome. Future preventative and therapeutic approaches very likely require a personalized combination of different targeting strategies based on specific genetic profiles and preclinical or clinical stages at the time of diagnosis and treatment.

## References

[1] Alzheimer’s Association (2018). 2018 Alzheimer’s disease facts and figures. Alzheimers Dement.

[2] Selkoe DJ (2001). Alzheimer’s disease: genes, proteins, and therapy. Physiol Rev.

[3] Lane CA, Hardy J, Schott JM (2018). Alzheimer’s disease. Eur J Neurol.

[4] Mattsson N, Schott JM, Hardy J, Turner MR, Zetterberg H (2016). Selective vulnerability in neurodegeneration: insights from clinical variants of Alzheimer’s disease. J Neurol Neurosurg Psychiatry.

[5] Raskin J, Cummings J, Hardy J, Schuh K, Dean RA (2015). Neurobiology of Alzheimer’s Disease: integrated molecular, Physiological, Anatomical, Biomarker, and Cognitive Dimensions. Curr Alzheimer Res.

[6] Fiest KM, Roberts JI, Maxwell CJ, Hogan DB, Smith EE, Frolkis A, Cohen A, Kirk A, Pearson D, Pringsheim T, Venegas-Torres A, Jette N (2016). The prevalence and incidence of dementia due to Alzheimer’s Disease: a systematic review and meta-analysis. Can J Neurol Sci.

[7] Jack CR, Bennett DA, Blennow K, Carrillo MC, Dunn B, Haeberlein SB, Holtzman DM, Jagust W, Jessen F, Karlawish J, Liu E, Molinuevo JL, Montine T, Phelps C, Rankin KP, Rowe CC, Scheltens P, Siemers E, Snyder HM, Sperling R, Contributors (2018). NIA-AA research framework: toward a biological definition of Alzheimer’s disease. Alzheimers Dement.

[8] Dubois B, Feldman HH, Jacova C, Dekosky ST, Barberger-Gateau P, Cummings J, Delacourte A, Galasko D, Gauthier S, Jicha G, Meguro K, O'Brien J, Pasquier F, Robert P, Rossor M, Salloway S, Stern Y, Visser PJ, Scheltens P (2007). Research criteria for the diagnosis of Alzheimer’s disease: revising the NINCDS-ADRDA criteria. Lancet Neurol.

[9] McKhann GM, Knopman DS, Chertkow H, Hyman BT, Jack CR, Kawas CH, Klunk WE, Koroshetz WJ, Manly JJ, Mayeux R, Mohs RC, Morris JC, Rossor MN, Scheltens P, Carrillo MC, Thies B, Weintraub S, Phelps CH (2011). The diagnosis of dementia due to Alzheimer’s disease: recommendations from the National Institute on Aging-Alzheimer’s Association workgroups on diagnostic guidelines for Alzheimer’s disease. Alzheimers Dement.

[10] Albert MS, DeKosky ST, Dickson D, Dubois B, Feldman HH, Fox NC, Gamst A, Holtzman DM, Jagust WJ, Petersen RC, Snyder PJ, Carrillo MC, Thies B, Phelps CH (2011). The diagnosis of mild cognitive impairment due to Alzheimer’s disease: recommendations from the National Institute on Aging-Alzheimer’s Association workgroups on diagnostic guidelines for Alzheimer’s disease. Alzheimers Dement.

[11] Thal DR, Rub U, Orantes M, Braak H (2002). Phases of A beta-deposition in the human brain and its relevance for the development of AD. Neurology.

[12] Serrano-Pozo A, Qian J, Muzikansky A, Monsell SE, Montine TJ, Frosch MP, Betensky RA, Hyman BT (2016). Thal amyloid stages do not significantly impact the correlation between neuropathological change and cognition in the Alzheimer Disease continuum. J Neuropathol Exp Neurol.

[13] Braak H, Braak E (1991). Neuropathological stageing of Alzheimer-related changes. Acta Neuropathol.

[14] Braak H, Braak E (1997). Frequency of stages of Alzheimer-related lesions in different age categories. Neurobiol Aging.

[15] Braak H, Del Tredici K (2018). Spreading of tau pathology in sporadic Alzheimer’s Disease along Cortico-cortical top-down connections. Cereb Cortex.

[16] Montine TJ, Monsell SE, Beach TG, Bigio EH, Bu Y, Cairns NJ, Frosch M, Henriksen J, Kofler J, Kukull WA, Lee EB, Nelson PT, Schantz AM, Schneider JA, Sonnen JA, Trojanowski JQ, Vinters HV, Zhou XH, Hyman BT (2016). Multisite assessment of NIA-AA guidelines for the neuropathologic evaluation of Alzheimer’s disease. Alzheimers Dement.

[17] Lagarde J, Sarazin M, Bottlaender M (2018). In vivo PET imaging of neuroinflammation in Alzheimer’s disease. J Neural Transm (Vienna).

[18] Veitch DP, Weiner MW, Aisen PS, Beckett LA, Cairns NJ, Green RC, Harvey D, Jack CR Jr, Jagust W, Morris JC, Petersen RC, Saykin AJ, Shaw LM, Toga AW, Trojanowski JQ, Alzheimer’s Disease Neuroimaging I. Understanding disease progression and improving Alzheimer’s disease clinical trials: recent highlights from the Alzheimer’s Disease Neuroimaging initiative. Alzheimers Dement. 2018. 10.1016/j.jalz.2018.08.005. 10.1016/j.jalz.2018.08.005 30321505

[19] Weiner MW, Veitch DP, Aisen PS, Beckett LA, Cairns NJ, Green RC, Harvey D, Jack CR, Jagust W, Morris JC, Petersen RC, Saykin AJ, Shaw LM, Toga AW, Trojanowski JQ, Alzheimer’s Disease Neuroimaging Initiative (2017). Recent publications from the Alzheimer’s Disease Neuroimaging initiative: reviewing progress toward improved AD clinical trials. Alzheimers Dement.

[20] Weiner MW, Veitch DP, Aisen PS, Beckett LA, Cairns NJ, Green RC, Harvey D, Jack CR, Jagust W, Morris JC, Petersen RC, Salazar J, Saykin AJ, Shaw LM, Toga AW, Trojanowski JQ, Alzheimer’s Disease Neuroimaging Initiative (2017). The Alzheimer’s Disease Neuroimaging initiative 3: continued innovation for clinical trial improvement. Alzheimers Dement.

[21] Rathore S, Habes M, Iftikhar MA, Shacklett A, Davatzikos C (2017). A review on neuroimaging-based classification studies and associated feature extraction methods for Alzheimer’s disease and its prodromal stages. Neuroimage.

[22] Villemagne VL, Chetelat G (2016). Neuroimaging biomarkers in Alzheimer’s disease and other dementias. Ageing Res Rev.

[23] Paraskevaidi M, Morais CLM, Freitas DLD, Lima KMG, Mann DMA, Allsop D, Martin-Hirsch PL, Martin FL. Blood-based near-infrared spectroscopy for the rapid low-cost detection of Alzheimer’s disease. Analyst. 2018. 10.1039/c8an01205a 30183030

[24] Tatebe H, Kasai T, Ohmichi T, Kishi Y, Kakeya T, Waragai M, Kondo M, Allsop D, Tokuda T (2017). Quantification of plasma phosphorylated tau to use as a biomarker for brain Alzheimer pathology: pilot case-control studies including patients with Alzheimer’s disease and Down syndrome. Mol Neurodegener.

[25] Jaeger PA, Lucin KM, Britschgi M, Vardarajan B, Huang RP, Kirby ED, Abbey R, Boeve BF, Boxer AL, Farrer LA, Finch N, Graff-Radford NR, Head E, Hoffree M, Huang R, Johns H, Karydas A, Knopman DS, Loboda A, Masliah E, Narasimhan R, Petersen RC, Podtelezhnikov A, Pradhan S, Rademakers R, Sun CH, Younkin SG, Miller BL, Ideker T, Wyss-Coray T (2016). Network-driven plasma proteomics expose molecular changes in the Alzheimer’s brain. Mol Neurodegener.

[26] Blennow K, Zetterberg H (2018). Biomarkers for Alzheimer disease - current status and prospects for the future. J Intern Med.

[27] O'Bryant SE, Gupta V, Henriksen K, Edwards M, Jeromin A, Lista S, Bazenet C, Soares H, Lovestone S, Hampel H, Montine T, Blennow K, Foroud T, Carrillo M, Graff-Radford N, Laske C, Breteler M, Shaw L, Trojanowski JQ, Schupf N, Rissman RA, Fagan AM, Oberoi P, Umek R, Weiner MW, Grammas P, Posner H, Martins R, Star B, Groups, B. w (2015). Guidelines for the standardization of preanalytic variables for blood-based biomarker studies in Alzheimer’s disease research. Alzheimers Dement.

[28] Olsson B, Lautner R, Andreasson U, Ohrfelt A, Portelius E, Bjerke M, Holtta M, Rosen C, Olsson C, Strobel G, Wu E, Dakin K, Petzold M, Blennow K, Zetterberg H (2016). CSF and blood biomarkers for the diagnosis of Alzheimer’s disease: a systematic review and meta-analysis. Lancet Neurol.

[29] Arneric SP, Batrla-Utermann R, Beckett L, Bittner T, Blennow K, Carter L, Dean R, Engelborghs S, Genius J, Gordon MF, Hitchcock J, Kaplow J, Luthman J, Meibach R, Raunig D, Romero K, Samtani MN, Savage M, Shaw L, Stephenson D, Umek RM, Vanderstichele H, Willis B, Yule S (2017). Cerebrospinal fluid biomarkers for Alzheimer’s Disease: A view of the regulatory science qualification landscape from the coalition against major diseases CSF biomarker team. J Alzheimers Dis.

[30] Blennow K (2017). A review of fluid biomarkers for Alzheimer’s Disease: Moving from CSF to blood. Neurol Ther.

[31] Blennow K, Zetterberg H (2018). The past and the future of Alzheimer’s Disease fluid biomarkers. J Alzheimers Dis.

[32] Sperling RA, Aisen PS, Beckett LA, Bennett DA, Craft S, Fagan AM, Iwatsubo T, Jack CR, Kaye J, Montine TJ, Park DC, Reiman EM, Rowe CC, Siemers E, Stern Y, Yaffe K, Carrillo MC, Thies B, Morrison-Bogorad M, Wagster MV, Phelps CH (2011). Toward defining the preclinical stages of Alzheimer’s disease: recommendations from the National Institute on Aging-Alzheimer’s Association workgroups on diagnostic guidelines for Alzheimer’s disease. Alzheimers Dement.

[33] Akiyama H, Barger S, Barnum S, Bradt B, Bauer J, Cole GM, Cooper NR, Eikelenboom P, Emmerling M, Fiebich BL, Finch CE, Frautschy S, Griffin WS, Hampel H, Hull M, Landreth G, Lue L, Mrak R, Mackenzie IR, McGeer PL, O'Banion MK, Pachter J, Pasinetti G, Plata-Salaman C, Rogers J, Rydel R, Shen Y, Streit W, Strohmeyer R, Tooyoma I, Van Muiswinkel FL, Veerhuis R, Walker D, Webster S, Wegrzyniak B, Wenk G, Wyss-Coray T (2000). Inflammation and Alzheimer’s disease. Neurobiol Aging.

[34] Ardura-Fabregat A, Boddeke E, Boza-Serrano A, Brioschi S, Castro-Gomez S, Ceyzeriat K, Dansokho C, Dierkes T, Gelders G, Heneka MT, Hoeijmakers L, Hoffmann A, Iaccarino L, Jahnert S, Kuhbandner K, Landreth G, Lonnemann N, Loschmann PA, McManus RM, Paulus A, Reemst K, Sanchez-Caro JM, Tiberi A, Van der Perren A, Vautheny A, Venegas C, Webers A, Weydt P, Wijasa TS, Xiang X, Yang Y (2017). Targeting Neuroinflammation to treat Alzheimer’s Disease. CNS Drugs.

[35] Heneka MT, Carson MJ, El Khoury J, Landreth GE, Brosseron F, Feinstein DL, Jacobs AH, Wyss-Coray T, Vitorica J, Ransohoff RM, Herrup K, Frautschy SA, Finsen B, Brown GC, Verkhratsky A, Yamanaka K, Koistinaho J, Latz E, Halle A, Petzold GC, Town T, Morgan D, Shinohara ML, Perry VH, Holmes C, Bazan NG, Brooks DJ, Hunot S, Joseph B, Deigendesch N, Garaschuk O, Boddeke E, Dinarello CA, Breitner JC, Cole GM, Golenbock DT, Kummer MP (2015). Neuroinflammation in Alzheimer’s disease. Lancet Neurol.

[36] Jay TR, von Saucken VE, Landreth GE (2017). TREM2 in neurodegenerative diseases. Mol Neurodegener.

[37] Zhao N, Liu CC, Qiao W, Bu G (2018). Apolipoprotein E, Receptors, and Modulation of Alzheimer’s Disease. Biol Psychiatry.

[38] Jiang T, Sun Q, Chen S (2016). Oxidative stress: A major pathogenesis and potential therapeutic target of antioxidative agents in Parkinson’s disease and Alzheimer’s disease. Prog Neurobiol.

[39] Paillusson S, Stoica R, Gomez-Suaga P, Lau DHW, Mueller S, Miller T, Miller CCJ (2016). There’s something wrong with my MAM; the ER-mitochondria Axis and neurodegenerative diseases. Trends Neurosci.

[40] Kerr JS, Adriaanse BA, Greig NH, Mattson MP, Cader MZ, Bohr VA, Fang EF (2017). Mitophagy and Alzheimer’s Disease: cellular and molecular mechanisms. Trends Neurosci.

[41] Iadecola C (2004). Neurovascular regulation in the normal brain and in Alzheimer’s disease. Nat Rev Neurosci.

[42] Iadecola C (2017). The neurovascular unit coming of age: A journey through neurovascular coupling in health and Disease. Neuron.

[43] Padurariu M, Ciobica A, Lefter R, Serban IL, Stefanescu C, Chirita R (2013). The oxidative stress hypothesis in Alzheimer’s disease. Psychiatr Danub.

[44] Pratico D (2008). Oxidative stress hypothesis in Alzheimer’s disease: a reappraisal. Trends Pharmacol Sci.

[45] Markesbery WR (1997). Oxidative stress hypothesis in Alzheimer’s disease. Free Radic Biol Med.

[46] Hopperton KE, Mohammad D, Trepanier MO, Giuliano V, Bazinet RP (2018). Markers of microglia in post-mortem brain samples from patients with Alzheimer’s disease: a systematic review. Mol Psychiatry.

[47] Bloom GS, Lazo JS, Norambuena A (2018). Reduced brain insulin signaling: A seminal process in Alzheimer’s disease pathogenesis. Neuropharmacology.

[48] Griffith CM, Eid T, Rose GM, Patrylo PR (2018). Evidence for altered insulin receptor signaling in Alzheimer’s disease. Neuropharmacology.

[49] Benedict C, Grillo CA (2018). Insulin resistance as a therapeutic target in the treatment of Alzheimer’s Disease: A state-of-the-art review. Front Neurosci.

[50] Hardy JA, Higgins GA (1992). Alzheimer’s disease: the amyloid cascade hypothesis. Science.

[51] Cleary JP, Walsh DM, Hofmeister JJ, Shankar GM, Kuskowski MA, Selkoe DJ, Ashe KH (2005). Natural oligomers of the amyloid-beta protein specifically disrupt cognitive function. Nat Neurosci.

[52] Shankar GM, Bloodgood BL, Townsend M, Walsh DM, Selkoe DJ, Sabatini BL (2007). Natural oligomers of the Alzheimer amyloid-beta protein induce reversible synapse loss by modulating an NMDA-type glutamate receptor-dependent signaling pathway. J Neurosci.

[53] Li S, Shankar GM, Selkoe DJ (2010). How do soluble oligomers of amyloid beta-protein impair hippocampal synaptic plasticity?. Front Cell Neurosci.

[54] Li S, Jin M, Koeglsperger T, Shepardson NE, Shankar GM, Selkoe DJ (2011). Soluble Abeta oligomers inhibit long-term potentiation through a mechanism involving excessive activation of extrasynaptic NR2B-containing NMDA receptors. J Neurosci.

[55] Mucke L, Selkoe DJ (2012). Neurotoxicity of amyloid beta-protein: synaptic and network dysfunction. Cold Spring Harb Perspect Med.

[56] Tu S, Okamoto S, Lipton SA, Xu H (2014). Oligomeric Abeta-induced synaptic dysfunction in Alzheimer’s disease. Mol Neurodegener.

[57] Musiek ES, Holtzman DM (2015). Three dimensions of the amyloid hypothesis: time, space and ’wingmen’. Nat Neurosci.

[58] Selkoe DJ, Hardy J (2016). The amyloid hypothesis of Alzheimer’s disease at 25 years. EMBO Mol Med.

[59] Du X, Wang X, Geng M (2018). Alzheimer’s disease hypothesis and related therapies. Transl Neurodegener.

[60] Morris GP, Clark IA, Vissel B (2018). Questions concerning the role of amyloid-beta in the definition, aetiology and diagnosis of Alzheimer’s disease. Acta Neuropathol.

[61] Wolfe MS, Xia W, Ostaszewski BL, Diehl TS, Kimberly WT, Selkoe DJ (1999). Two transmembrane aspartates in presenilin-1 required for presenilin endoproteolysis and gamma-secretase activity. Nature.

[62] Bettens K, Sleegers K, Van Broeckhoven C (2013). Genetic insights in Alzheimer’s disease. Lancet Neurol.

[63] De Strooper B, Saftig P, Craessaerts K, Vanderstichele H, Guhde G, Annaert W, Von Figura K, Van Leuven F (1998). Deficiency of presenilin-1 inhibits the normal cleavage of amyloid precursor protein. Nature.

[64] Bateman RJ, Aisen PS, De Strooper B, Fox NC, Lemere CA, Ringman JM, Salloway S, Sperling RA, Windisch M, Xiong C (2011). Autosomal-dominant Alzheimer’s disease: a review and proposal for the prevention of Alzheimer’s disease. Alzheimers Res Ther.

[65] Mawuenyega KG, Sigurdson W, Ovod V, Munsell L, Kasten T, Morris JC, Yarasheski KE, Bateman RJ (2010). Decreased clearance of CNS beta-amyloid in Alzheimer’s disease. Science.

[66] Castellano JM, Kim J, Stewart FR, Jiang H, DeMattos RB, Patterson BW, Fagan AM, Morris JC, Mawuenyega KG, Cruchaga C, Goate AM, Bales KR, Paul SM, Bateman RJ, Holtzman DM (2011). Human apoE isoforms differentially regulate brain amyloid-beta peptide clearance. Sci Transl Med.

[67] Hu J, Liu CC, Chen XF, Zhang YW, Xu H, Bu G (2015). Opposing effects of viral mediated brain expression of apolipoprotein E2 (apoE2) and apoE4 on apoE lipidation and Abeta metabolism in apoE4-targeted replacement mice. Mol Neurodegener.

[68] Liu CC, Zhao N, Fu Y, Wang N, Linares C, Tsai CW, Bu G (2017). ApoE4 accelerates early seeding of amyloid pathology. Neuron.

[69] Terry RD, Masliah E, Salmon DP, Butters N, DeTeresa R, Hill R, Hansen LA, Katzman R (1991). Physical basis of cognitive alterations in Alzheimer’s disease: synapse loss is the major correlate of cognitive impairment. Ann Neurol.

[70] Lambert MP, Barlow AK, Chromy BA, Edwards C, Freed R, Liosatos M, Morgan TE, Rozovsky I, Trommer B, Viola KL, Wals P, Zhang C, Finch CE, Krafft GA, Klein WL (1998). Diffusible, nonfibrillar ligands derived from Abeta1-42 are potent central nervous system neurotoxins. Proc Natl Acad Sci U S A.

[71] Forner S, Baglietto-Vargas D, Martini AC, Trujillo-Estrada L, LaFerla FM (2017). Synaptic impairment in Alzheimer’s Disease: A dysregulated symphony. Trends Neurosci.

[72] Audrain M, Fol R, Dutar P, Potier B, Billard JM, Flament J, Alves S, Burlot MA, Dufayet-Chaffaud G, Bemelmans AP, Valette J, Hantraye P, Deglon N, Cartier N, Braudeau J (2016). Alzheimer’s disease-like APP processing in wild-type mice identifies synaptic defects as initial steps of disease progression. Mol Neurodegener.

[73] Abramov AY, Canevari L, Duchen MR (2004). Calcium signals induced by amyloid beta peptide and their consequences in neurons and astrocytes in culture. Biochim Biophys Acta.

[74] Canevari L, Abramov AY, Duchen MR (2004). Toxicity of amyloid beta peptide: tales of calcium, mitochondria, and oxidative stress. Neurochem Res.

[75] Camandola S, Mattson MP (2011). Aberrant subcellular neuronal calcium regulation in aging and Alzheimer’s disease. Biochim Biophys Acta.

[76] Mattson MP (2010). ER calcium and Alzheimer’s disease: in a state of flux. Sci Signal.

[77] Ferrarelli LK. New connections: amyloid-beta, calcium, and the synapse. Sci Signal. 2017;10. 10.1126/scisignal.aao3024 28698217

[78] Arbel-Ornath M, Hudry E, Boivin JR, Hashimoto T, Takeda S, Kuchibhotla KV, Hou S, Lattarulo CR, Belcher AM, Shakerdge N, Trujillo PB, Muzikansky A, Betensky RA, Hyman BT, Bacskai BJ (2017). Soluble oligomeric amyloid-beta induces calcium dyshomeostasis that precedes synapse loss in the living mouse brain. Mol Neurodegener.

[79] Demuro A, Parker I, Stutzmann GE (2010). Calcium signaling and amyloid toxicity in Alzheimer disease. J Biol Chem.

[80] Bai Y, Li M, Zhou Y, Ma L, Qiao Q, Hu W, Li W, Wills ZP, Gan WB (2017). Abnormal dendritic calcium activity and synaptic depotentiation occur early in a mouse model of Alzheimer’s disease. Mol Neurodegener.

[81] Hiruma H, Katakura T, Takahashi S, Ichikawa T, Kawakami T (2003). Glutamate and amyloid beta-protein rapidly inhibit fast axonal transport in cultured rat hippocampal neurons by different mechanisms. J Neurosci.

[82] Pigino G, Morfini G, Atagi Y, Deshpande A, Yu C, Jungbauer L, LaDu M, Busciglio J, Brady S (2009). Disruption of fast axonal transport is a pathogenic mechanism for intraneuronal amyloid beta. Proc Natl Acad Sci U S A.

[83] Decker H, Lo KY, Unger SM, Ferreira ST, Silverman MA (2010). Amyloid-beta peptide oligomers disrupt axonal transport through an NMDA receptor-dependent mechanism that is mediated by glycogen synthase kinase 3beta in primary cultured hippocampal neurons. J Neurosci.

[84] Deng M, He W, Tan Y, Han H, Hu X, Xia K, Zhang Z, Yan R (2013). Increased expression of reticulon 3 in neurons leads to reduced axonal transport of beta site amyloid precursor protein-cleaving enzyme 1. J Biol Chem.

[85] Choi H, Kim HJ, Kim J, Kim S, Yang J, Lee W, Park Y, Hyeon SJ, Lee DS, Ryu H, Chung J, Mook-Jung I (2017). Increased acetylation of Peroxiredoxin1 by HDAC6 inhibition leads to recovery of Abeta-induced impaired axonal transport. Mol Neurodegener.

[86] Liu RQ, Zhou QH, Ji SR, Zhou Q, Feng D, Wu Y, Sui SF (2010). Membrane localization of beta-amyloid 1-42 in lysosomes: a possible mechanism for lysosome labilization. J Biol Chem.

[87] Volgyi K, Juhasz G, Kovacs Z, Penke B (2015). Dysfunction of endoplasmic reticulum (ER) and mitochondria (MT) in Alzheimer’s Disease: the role of the ER-MT cross-talk. Curr Alzheimer Res.

[88] Sun X, Chen WD, Wang YD (2015). Beta-amyloid: the key peptide in the pathogenesis of Alzheimer’s disease. Front Pharmacol.

[89] Joshi G, Chi Y, Huang Z, Wang Y (2014). Abeta-induced Golgi fragmentation in Alzheimer’s disease enhances Abeta production. Proc Natl Acad Sci U S A.

[90] Joshi G, Bekier ME, Wang Y (2015). Golgi fragmentation in Alzheimer’s disease. Front Neurosci.

[91] Sollvander S, Nikitidou E, Brolin R, Soderberg L, Sehlin D, Lannfelt L, Erlandsson A (2016). Accumulation of amyloid-beta by astrocytes result in enlarged endosomes and microvesicle-induced apoptosis of neurons. Mol Neurodegener.

[92] Butterfield DA, Boyd-Kimball D (2018). Oxidative stress, Amyloid-beta Peptide, and Altered Key Molecular Pathways in the Pathogenesis and Progression of Alzheimer’s Disease. J Alzheimers Dis.

[93] Willen K, Edgar JR, Hasegawa T, Tanaka N, Futter CE, Gouras GK (2017). Abeta accumulation causes MVB enlargement and is modelled by dominant negative VPS4A. Mol Neurodegener.

[94] Park J, Choi H, Min JS, Kim B, Lee SR, Yun JW, Choi MS, Chang KT, Lee DS (2015). Loss of mitofusin 2 links beta-amyloid-mediated mitochondrial fragmentation and Cdk5-induced oxidative stress in neuron cells. J Neurochem.

[95] Jiang S, Nandy P, Wang W, Ma X, Hsia J, Wang C, Wang Z, Niu M, Siedlak SL, Torres S, Fujioka H, Xu Y, Lee HG, Perry G, Liu J, Zhu X (2018). Mfn2 ablation causes an oxidative stress response and eventual neuronal death in the hippocampus and cortex. Mol Neurodegener.

[96] Rodriguez-Vieitez E, Saint-Aubert L, Carter SF, Almkvist O, Farid K, Scholl M, Chiotis K, Thordardottir S, Graff C, Wall A, Langstrom B, Nordberg A (2016). Diverging longitudinal changes in astrocytosis and amyloid PET in autosomal dominant Alzheimer’s disease. Brain.

[97] Rodriguez-Vieitez E, Ni R, Gulyas B, Toth M, Haggkvist J, Halldin C, Voytenko L, Marutle A, Nordberg A (2015). Astrocytosis precedes amyloid plaque deposition in Alzheimer APPswe transgenic mouse brain: a correlative positron emission tomography and in vitro imaging study. Eur J Nucl Med Mol Imaging.

[98] Pike CJ, Cummings BJ, Monzavi R, Cotman CW (1994). Beta-amyloid-induced changes in cultured astrocytes parallel reactive astrocytosis associated with senile plaques in Alzheimer’s disease. Neuroscience.

[99] Craft JM, Watterson DM, Van Eldik LJ (2006). Human amyloid beta-induced neuroinflammation is an early event in neurodegeneration. Glia.

[100] Cai Z, Hussain MD, Yan LJ (2014). Microglia, neuroinflammation, and beta-amyloid protein in Alzheimer’s disease. Int J Neurosci.

[101] Futch HS, Croft CL, Truong VQ, Krause EG, Golde TE (2017). Targeting psychologic stress signaling pathways in Alzheimer’s disease. Mol Neurodegener.

[102] Efthymiou AG, Goate AM (2017). Late onset Alzheimer’s disease genetics implicates microglial pathways in disease risk. Mol Neurodegener.

[103] Saresella M, La Rosa F, Piancone F, Zoppis M, Marventano I, Calabrese E, Rainone V, Nemni R, Mancuso R, Clerici M (2016). The NLRP3 and NLRP1 inflammasomes are activated in Alzheimer’s disease. Mol Neurodegener.

[104] Jankowsky JL, Zheng H (2017). Practical considerations for choosing a mouse model of Alzheimer’s disease. Mol Neurodegener.

[105] LaFerla FM, Green KN. Animal models of Alzheimer disease. Cold Spring Harb Perspect Med. 2012;2. 10.1101/cshperspect.a006320 PMC3543097 23002015

[106] Guo Q, Wang Z, Li H, Wiese M, Zheng H (2012). APP physiological and pathophysiological functions: insights from animal models. Cell Res.

[107] Stancu IC, Vasconcelos B, Terwel D, Dewachter I (2014). Models of beta-amyloid induced tau-pathology: the long and “folded” road to understand the mechanism. Mol Neurodegener.

[108] He Z, Guo JL, McBride JD, Narasimhan S, Kim H, Changolkar L, Zhang B, Gathagan RJ, Yue C, Dengler C, Stieber A, Nitla M, Coulter DA, Abel T, Brunden KR, Trojanowski JQ, Lee VM (2018). Amyloid-beta plaques enhance Alzheimer’s brain tau-seeded pathologies by facilitating neuritic plaque tau aggregation. Nat Med.

[109] Cohen RM, Rezai-Zadeh K, Weitz TM, Rentsendorj A, Gate D, Spivak I, Bholat Y, Vasilevko V, Glabe CG, Breunig JJ, Rakic P, Davtyan H, Agadjanyan MG, Kepe V, Barrio JR, Bannykh S, Szekely CA, Pechnick RN, Town T (2013). A transgenic Alzheimer rat with plaques, tau pathology, behavioral impairment, oligomeric abeta, and frank neuronal loss. J Neurosci.

[110] Forny-Germano L, e Silva NM, Batista AF, Brito-Moreira J, Gralle M, Boehnke SE, Coe BC, Lablans A, Marques SA, Martinez AM, Klein WL, Houzel JC, Ferreira ST, Munoz DP, De Felice FG (2014). Alzheimer’s disease-like pathology induced by amyloid-beta oligomers in nonhuman primates. J Neurosci.

[111] Choi SH, Kim YH, Hebisch M, Sliwinski C, Lee S, D'Avanzo C, Chen H, Hooli B, Asselin C, Muffat J, Klee JB, Zhang C, Wainger BJ, Peitz M, Kovacs DM, Woolf CJ, Wagner SL, Tanzi RE, Kim DY (2014). A three-dimensional human neural cell culture model of Alzheimer’s disease. Nature.

[112] Kim YH, Choi SH, D'Avanzo C, Hebisch M, Sliwinski C, Bylykbashi E, Washicosky KJ, Klee JB, Brustle O, Tanzi RE, Kim DY (2015). A 3D human neural cell culture system for modeling Alzheimer’s disease. Nat Protoc.

[113] Choi SH, Kim YH, Quinti L, Tanzi RE, Kim DY (2016). 3D culture models of Alzheimer’s disease: a road map to a “cure-in-a-dish”. Mol Neurodegener.

[114] Yang J, Li S, He XB, Cheng C, Le W (2016). Induced pluripotent stem cells in Alzheimer’s disease: applications for disease modeling and cell-replacement therapy. Mol Neurodegener.

[115] Espuny-Camacho I, Arranz AM, Fiers M, Snellinx A, Ando K, Munck S, Bonnefont J, Lambot L, Corthout N, Omodho L, Eynden EV, Radaelli E, Tesseur I, Wray S, Ebneth A, Hardy J, Leroy K, Brion JP, Vanderhaeghen P, De Strooper B (2017). Hallmarks of Alzheimer’s Disease in stem-cell-derived human neurons transplanted into mouse brain. Neuron.

[116] Ricciarelli R, Fedele E (2017). The amyloid Cascade hypothesis in Alzheimer’s Disease: It’s time to change our mind. Curr Neuropharmacol.

[117] Karran E, De Strooper B (2016). The amyloid cascade hypothesis: are we poised for success or failure?. J Neurochem.

[118] Harrison JR, Owen MJ (2016). Alzheimer’s disease: the amyloid hypothesis on trial. Br J Psychiatry.

[119] Naslund J, Haroutunian V, Mohs R, Davis KL, Davies P, Greengard P, Buxbaum JD (2000). Correlation between elevated levels of amyloid beta-peptide in the brain and cognitive decline. JAMA.

[120] Arriagada PV, Growdon JH, Hedley-Whyte ET, Hyman BT (1992). Neurofibrillary tangles but not senile plaques parallel duration and severity of Alzheimer’s disease. Neurology.

[121] Rodrigue KM, Kennedy KM, Devous MD, Rieck JR, Hebrank AC, Diaz-Arrastia R, Mathews D, Park DC (2012). Beta-amyloid burden in healthy aging: regional distribution and cognitive consequences. Neurology.

[122] Katzman R, Terry R, DeTeresa R, Brown T, Davies P, Fuld P, Renbing X, Peck A (1988). Clinical, pathological, and neurochemical changes in dementia: a subgroup with preserved mental status and numerous neocortical plaques. Ann Neurol.

[123] Dickson DW, Crystal HA, Mattiace LA, Masur DM, Blau AD, Davies P, Yen SH, Aronson MK (1992). Identification of normal and pathological aging in prospectively studied nondemented elderly humans. Neurobiol Aging.

[124] Aizenstein HJ, Nebes RD, Saxton JA, Price JC, Mathis CA, Tsopelas ND, Ziolko SK, James JA, Snitz BE, Houck PR, Bi W, Cohen AD, Lopresti BJ, DeKosky ST, Halligan EM, Klunk WE (2008). Frequent amyloid deposition without significant cognitive impairment among the elderly. Arch Neurol.

[125] Josephs KA, Whitwell JL, Ahmed Z, Shiung MM, Weigand SD, Knopman DS, Boeve BF, Parisi JE, Petersen RC, Dickson DW, Jack CR (2008). Beta-amyloid burden is not associated with rates of brain atrophy. Ann Neurol.

[126] Murray ME, Dickson DW (2014). Is pathological aging a successful resistance against amyloid-beta or preclinical Alzheimer’s disease?. Alzheimers Res Ther.

[127] Cummings J, Lee G, Ritter A, Zhong K (2018). Alzheimer’s disease drug development pipeline: 2018. Alzheimers Dement (N Y).

[128] Cummings J (2018). Lessons learned from Alzheimer Disease: clinical trials with negative outcomes. Clin Transl Sci.

[129] Walsh DM, Selkoe DJ (2016). A critical appraisal of the pathogenic protein spread hypothesis of neurodegeneration. Nat Rev Neurosci.

[130] Selkoe DJ (2013). The therapeutics of Alzheimer’s disease: where we stand and where we are heading. Ann Neurol.

[131] Malek-Ahmadi M, Perez SE, Chen K, Mufson EJ (2016). Neuritic and diffuse plaque associations with memory in non-cognitively impaired elderly. J Alzheimers Dis.

[132] Tiraboschi P, Hansen LA, Thal LJ, Corey-Bloom J (2004). The importance of neuritic plaques and tangles to the development and evolution of AD. Neurology.

[133] Duyckaerts C, Colle MA, Dessi F, Piette F, Hauw JJ (1998). Progression of Alzheimer histopathological changes. Acta Neurol Belg.

[134] Giannakopoulos P, Herrmann FR, Bussiere T, Bouras C, Kovari E, Perl DP, Morrison JH, Gold G, Hof PR (2003). Tangle and neuron numbers, but not amyloid load, predict cognitive status in Alzheimer’s disease. Neurology.

[135] Ingelsson M, Fukumoto H, Newell KL, Growdon JH, Hedley-Whyte ET, Frosch MP, Albert MS, Hyman BT, Irizarry MC (2004). Early Abeta accumulation and progressive synaptic loss, gliosis, and tangle formation in AD brain. Neurology.

[136] Sarazin M, Lagarde J, Bottlaender M (2016). Distinct tau PET imaging patterns in typical and atypical Alzheimer’s disease. Brain.

[137] Thal DR, Vandenberghe R (2016). Monitoring the progression of Alzheimer’s disease with tau-PET. Brain.

[138] Ossenkoppele R, Schonhaut DR, Scholl M, Lockhart SN, Ayakta N, Baker SL, O'Neil JP, Janabi M, Lazaris A, Cantwell A, Vogel J, Santos M, Miller ZA, Bettcher BM, Vossel KA, Kramer JH, Gorno-Tempini ML, Miller BL, Jagust WJ, Rabinovici GD (2016). Tau PET patterns mirror clinical and neuroanatomical variability in Alzheimer’s disease. Brain.

[139] Scholl M, Lockhart SN, Schonhaut DR, O'Neil JP, Janabi M, Ossenkoppele R, Baker SL, Vogel JW, Faria J, Schwimmer HD, Rabinovici GD, Jagust WJ (2016). PET imaging of tau deposition in the aging human brain. Neuron.

[140] Schwarz AJ, Yu P, Miller BB, Shcherbinin S, Dickson J, Navitsky M, Joshi AD, Devous MD, Mintun MS (2016). Regional profiles of the candidate tau PET ligand 18F-AV-1451 recapitulate key features of Braak histopathological stages. Brain.

[141] Saint-Aubert L, Lemoine L, Chiotis K, Leuzy A, Rodriguez-Vieitez E, Nordberg A (2017). Tau PET imaging: present and future directions. Mol Neurodegener.

[142] Bennett DA, Schneider JA, Arvanitakis Z, Kelly JF, Aggarwal NT, Shah RC, Wilson RS (2006). Neuropathology of older persons without cognitive impairment from two community-based studies. Neurology.

[143] Price JL, McKeel DW, Buckles VD, Roe CM, Xiong C, Grundman M, Hansen LA, Petersen RC, Parisi JE, Dickson DW, Smith CD, Davis DG, Schmitt FA, Markesbery WR, Kaye J, Kurlan R, Hulette C, Kurland BF, Higdon R, Kukull W, Morris JC (2009). Neuropathology of nondemented aging: presumptive evidence for preclinical Alzheimer disease. Neurobiol Aging.

[144] van Rossum IA, Visser PJ, Knol DL, van der Flier WM, Teunissen CE, Barkhof F, Blankenstein MA, Scheltens P (2012). Injury markers but not amyloid markers are associated with rapid progression from mild cognitive impairment to dementia in Alzheimer’s disease. J Alzheimers Dis.

[145] van Rossum IA, Vos SJ, Burns L, Knol DL, Scheltens P, Soininen H, Wahlund LO, Hampel H, Tsolaki M, Minthon L, L'Italien G, van der Flier WM, Teunissen CE, Blennow K, Barkhof F, Rueckert D, Wolz R, Verhey F, Visser PJ (2012). Injury markers predict time to dementia in subjects with MCI and amyloid pathology. Neurology.

[146] Rapoport M, Dawson HN, Binder LI, Vitek MP, Ferreira A (2002). Tau is essential to beta -amyloid-induced neurotoxicity. Proc Natl Acad Sci U S A.

[147] Roberson ED, Scearce-Levie K, Palop JJ, Yan F, Cheng IH, Wu T, Gerstein H, Yu GQ, Mucke L (2007). Reducing endogenous tau ameliorates amyloid beta-induced deficits in an Alzheimer’s disease mouse model. Science.

[148] Shipton OA, Leitz JR, Dworzak J, Acton CE, Tunbridge EM, Denk F, Dawson HN, Vitek MP, Wade-Martins R, Paulsen O, Vargas-Caballero M (2011). Tau protein is required for amyloid {beta}-induced impairment of hippocampal long-term potentiation. J Neurosci.

[149] DeVos SL, Corjuc BT, Commins C, Dujardin S, Bannon RN, Corjuc D, Moore BD, Bennett RE, Jorfi M, Gonzales JA, Dooley PM, Roe AD, Pitstick R, Irimia D, Frosch MP, Carlson GA, Hyman BT (2018). Tau reduction in the presence of amyloid-beta prevents tau pathology and neuronal death in vivo. Brain.

[150] DeVos SL, Miller RL, Schoch KM, Holmes BB, Kebodeaux CS, Wegener AJ, Chen G, Shen T, Tran H, Nichols B, Zanardi TA, Kordasiewicz HB, Swayze EE, Bennett CF, Diamond MI, Miller TM. Tau reduction prevents neuronal loss and reverses pathological tau deposition and seeding in mice with tauopathy. Sci Transl Med. 2017;9. 10.1126/scitranslmed.aag0481 PMC5792300 28123067

[151] Vossel KA, Xu JC, Fomenko V, Miyamoto T, Suberbielle E, Knox JA, Ho K, Kim DH, Yu GQ, Mucke L (2015). Tau reduction prevents Abeta-induced axonal transport deficits by blocking activation of GSK3beta. J Cell Biol.

[152] Ittner LM, Ke YD, Delerue F, Bi M, Gladbach A, van Eersel J, Wölfing H, Chieng BC, Christie MJ, Napier IA, Eckert A, Staufenbiel M, Hardeman E, Götz J. Dendritic function of tau mediates amyloid-beta toxicity in Alzheimer's disease mouse models. Cell. 2010;142:387–97. 10.1016/j.cell.2010.06.036 20655099

[153] Vossel KA, Zhang K, Brodbeck J, Daub AC, Sharma P, Finkbeiner S, Cui B, Mucke L (2010). Tau reduction prevents Abeta-induced defects in axonal transport. Science.

[154] Wang Y, Mandelkow E (2016). Tau in physiology and pathology. Nat Rev Neurosci.

[155] Spillantini MG, Goedert M (2013). Tau pathology and neurodegeneration. Lancet Neurol.

[156] Iqbal K, Liu F, Gong CX, Alonso Adel C, Grundke-Iqbal I (2009). Mechanisms of tau-induced neurodegeneration. Acta Neuropathol.

[157] Guo T, Noble W, Hanger DP (2017). Roles of tau protein in health and disease. Acta Neuropathol.

[158] Morris M, Knudsen GM, Maeda S, Trinidad JC, Ioanoviciu A, Burlingame AL, Mucke L (2015). Tau post-translational modifications in wild-type and human amyloid precursor protein transgenic mice. Nat Neurosci.

[159] Wu XL, Pina-Crespo J, Zhang YW, Chen XC, Xu HX (2017). Tau-mediated neurodegeneration and potential implications in diagnosis and treatment of Alzheimer’s Disease. Chin Med J.

[160] Sotiropoulos I, Galas MC, Silva JM, Skoulakis E, Wegmann S, Maina MB, Blum D, Sayas CL, Mandelkow EM, Mandelkow E, Spillantini MG, Sousa N, Avila J, Medina M, Mudher A, Buee L (2017). Atypical, non-standard functions of the microtubule associated tau protein. Acta Neuropathol Commun.

[161] Pooler AM, Noble W, Hanger DP (2014). A role for tau at the synapse in Alzheimer’s disease pathogenesis. Neuropharmacology.

[162] Violet M, Chauderlier A, Delattre L, Tardivel M, Chouala MS, Sultan A, Marciniak E, Humez S, Binder L, Kayed R, Lefebvre B, Bonnefoy E, Buee L, Galas MC (2015). Prefibrillar tau oligomers alter the nucleic acid protective function of tau in hippocampal neurons in vivo. Neurobiol Dis.

[163] Sultan A, Nesslany F, Violet M, Begard S, Loyens A, Talahari S, Mansuroglu Z, Marzin D, Sergeant N, Humez S, Colin M, Bonnefoy E, Buee L, Galas MC (2011). Nuclear tau, a key player in neuronal DNA protection. J Biol Chem.

[164] Eftekharzadeh B, Daigle JG, Kapinos LE, Coyne A, Schiantarelli J, Carlomagno Y, Cook C, Miller SJ, Dujardin S, Amaral AS, Grima JC, Bennett RE, Tepper K, DeTure M, Vanderburgh CR, Corjuc BT, DeVos SL, Gonzalez JA, Chew J, Vidensky S, Gage FH, Mertens J, Troncoso J, Mandelkow E, Salvatella X, Lim RYH, Petrucelli L, Wegmann S, Rothstein JD, Hyman BT (2018). Tau protein disrupts nucleocytoplasmic transport in Alzheimer’s Disease. Neuron.

[165] Hu W, Zhang X, Tung YC, Xie S, Liu F, Iqbal K (2016). Hyperphosphorylation determines both the spread and the morphology of tau pathology. Alzheimers Dement.

[166] Gong CX, Iqbal K (2008). Hyperphosphorylation of microtubule-associated protein tau: a promising therapeutic target for Alzheimer disease. Curr Med Chem.

[167] Mazanetz MP, Fischer PM (2007). Untangling tau hyperphosphorylation in drug design for neurodegenerative diseases. Nat Rev Drug Discov.

[168] Cook C, Stankowski JN, Carlomagno Y, Stetler C, Petrucelli L (2014). Acetylation: a new key to unlock tau’s role in neurodegeneration. Alzheimers Res Ther.

[169] Min SW, Chen X, Tracy TE, Li Y, Zhou Y, Wang C, Shirakawa K, Minami SS, Defensor E, Mok SA, Sohn PD, Schilling B, Cong X, Ellerby L, Gibson BW, Johnson J, Krogan N, Shamloo M, Gestwicki J, Masliah E, Verdin E, Gan L (2015). Critical role of acetylation in tau-mediated neurodegeneration and cognitive deficits. Nat Med.

[170] Tracy TE, Gan L. Acetylated tau in Alzheimer’s disease: An instigator of synaptic dysfunction underlying memory loss: Increased levels of acetylated tau blocks the postsynaptic signaling required for plasticity and promotes memory deficits associated with tauopathy. Bioessays. 2017;39. 10.1002/bies.201600224 PMC5903676 28083916

[171] Park S, Lee JH, Jeon JH, Lee MJ (2018). Degradation or aggregation: the ramifications of post-translational modifications on tau. BMB Rep.

[172] Gong CX, Liu F, Iqbal K (2016). O-GlcNAcylation: A regulator of tau pathology and neurodegeneration. Alzheimers Dement.

[173] Zheng BW, Yang L, Dai XL, Jiang ZF, Huang HC (2016). Roles of O-GlcNAcylation on amyloid-beta precursor protein processing, tau phosphorylation, and hippocampal synapses dysfunction in Alzheimer’s disease. Neurol Res.

[174] Hastings NB, Wang X, Song L, Butts BD, Grotz D, Hargreaves R, Fred Hess J, Hong KK, Huang CR, Hyde L, Laverty M, Lee J, Levitan D, Lu SX, Maguire M, Mahadomrongkul V, McEachern EJ, Ouyang X, Rosahl TW, Selnick H, Stanton M, Terracina G, Vocadlo DJ, Wang G, Duffy JL, Parker EM, Zhang L (2017). Inhibition of O-GlcNAcase leads to elevation of O-GlcNAc tau and reduction of tauopathy and cerebrospinal fluid tau in rTg4510 mice. Mol Neurodegener.

[175] Sohn PD, Tracy TE, Son HI, Zhou Y, Leite RE, Miller BL, Seeley WW, Grinberg LT, Gan L (2016). Acetylated tau destabilizes the cytoskeleton in the axon initial segment and is mislocalized to the somatodendritic compartment. Mol Neurodegener.

[176] Wang YP, Biernat J, Pickhardt M, Mandelkow E, Mandelkow EM (2007). Stepwise proteolysis liberates tau fragments that nucleate the Alzheimer-like aggregation of full-length tau in a neuronal cell model. Proc Natl Acad Sci U S A.

[177] Wang Y, Garg S, Mandelkow EM, Mandelkow E (2010). Proteolytic processing of tau. Biochem Soc Trans.

[178] de Calignon A, Fox LM, Pitstick R, Carlson GA, Bacskai BJ, Spires-Jones TL, Hyman BT (2010). Caspase activation precedes and leads to tangles. Nature.

[179] Zhang Z, Song M, Liu X, Kang SS, Kwon IS, Duong DM, Seyfried NT, Hu WT, Liu Z, Wang JZ, Cheng L, Sun YE, Yu SP, Levey AI, Ye K (2014). Cleavage of tau by asparagine endopeptidase mediates the neurofibrillary pathology in Alzheimer’s disease. Nat Med.

[180] Petrucelli L, Dickson D, Kehoe K, Taylor J, Snyder H, Grover A, De Lucia M, McGowan E, Lewis J, Prihar G, Kim J, Dillmann WH, Browne SE, Hall A, Voellmy R, Tsuboi Y, Dawson TM, Wolozin B, Hardy J, Hutton M (2004). CHIP and Hsp70 regulate tau ubiquitination, degradation and aggregation. Hum Mol Genet.

[181] Cripps D, Thomas SN, Jeng Y, Yang F, Davies P, Yang AJ (2006). Alzheimer disease-specific conformation of hyperphosphorylated paired helical filament-tau is polyubiquitinated through Lys-48, Lys-11, and Lys-6 ubiquitin conjugation. J Biol Chem.

[182] Luo HB, Xia YY, Shu XJ, Liu ZC, Feng Y, Liu XH, Yu G, Yin G, Xiong YS, Zeng K, Jiang J, Ye K, Wang XC, Wang JZ (2014). SUMOylation at K340 inhibits tau degradation through deregulating its phosphorylation and ubiquitination. Proc Natl Acad Sci U S A.

[183] Reynolds MR, Reyes JF, Fu Y, Bigio EH, Guillozet-Bongaarts AL, Berry RW, Binder LI (2006). Tau nitration occurs at tyrosine 29 in the fibrillar lesions of Alzheimer’s disease and other tauopathies. J Neurosci.

[184] Reyes JF, Fu Y, Vana L, Kanaan NM, Binder LI (2011). Tyrosine nitration within the proline-rich region of tau in Alzheimer’s disease. Am J Pathol.

[185] Reyes JF, Geula C, Vana L, Binder LI (2012). Selective tau tyrosine nitration in non-AD tauopathies. Acta Neuropathol.

[186] Chu D, Liu F. Pathological changes of tau related to Alzheimer’s Disease. ACS Chem Neurosci. 2018. 10.1021/acschemneuro.8b00457 30346708

[187] Zhou Y, Shi J, Chu D, Hu W, Guan Z, Gong CX, Iqbal K, Liu F (2018). Relevance of phosphorylation and truncation of tau to the Etiopathogenesis of Alzheimer’s Disease. Front Aging Neurosci.

[188] Cheng Y, Bai F (2018). The Association of tau with Mitochondrial Dysfunction in Alzheimer’s Disease. Front Neurosci.

[189] Li XC, Hu Y, Wang ZH, Luo Y, Zhang Y, Liu XP, Feng Q, Wang Q, Ye K, Liu GP, Wang JZ (2016). Human wild-type full-length tau accumulation disrupts mitochondrial dynamics and the functions via increasing mitofusins. Sci Rep.

[190] Kopeikina KJ, Carlson GA, Pitstick R, Ludvigson AE, Peters A, Luebke JI, Koffie RM, Frosch MP, Hyman BT, Spires-Jones TL (2011). Tau accumulation causes mitochondrial distribution deficits in neurons in a mouse model of tauopathy and in human Alzheimer’s disease brain. Am J Pathol.

[191] Reddy PH (2011). Abnormal tau, mitochondrial dysfunction, impaired axonal transport of mitochondria, and synaptic deprivation in Alzheimer’s disease. Brain Res.

[192] Kandimalla R, Manczak M, Fry D, Suneetha Y, Sesaki H, Reddy PH (2016). Reduced dynamin-related protein 1 protects against phosphorylated tau-induced mitochondrial dysfunction and synaptic damage in Alzheimer’s disease. Hum Mol Genet.

[193] Boehm J (2013). A ’danse macabre’: tau and Fyn in STEP with amyloid beta to facilitate induction of synaptic depression and excitotoxicity. Eur J Neurosci.

[194] Crimins JL, Pooler A, Polydoro M, Luebke JI, Spires-Jones TL (2013). The intersection of amyloid beta and tau in glutamatergic synaptic dysfunction and collapse in Alzheimer’s disease. Ageing Res Rev.

[195] Liao D, Miller EC, Teravskis PJ (2014). Tau acts as a mediator for Alzheimer’s disease-related synaptic deficits. Eur J Neurosci.

[196] Miyamoto T, Stein L, Thomas R, Djukic B, Taneja P, Knox J, Vossel K, Mucke L (2017). Phosphorylation of tau at Y18, but not tau-fyn binding, is required for tau to modulate NMDA receptor-dependent excitotoxicity in primary neuronal culture. Mol Neurodegener.

[197] Talmat-Amar Y, Arribat Y, Parmentier ML. Vesicular Axonal Transport is Modified In Vivo by Tau Deletion or Overexpression in Drosophila. Int J Mol Sci. 2018;19. 10.3390/ijms19030744 PMC5877605 29509687

[198] Lacovich V, Espindola SL, Alloatti M, Pozo Devoto V, Cromberg LE, Carna ME, Forte G, Gallo JM, Bruno L, Stokin GB, Avale ME, Falzone TL (2017). Tau isoforms imbalance impairs the axonal transport of the amyloid precursor protein in human neurons. J Neurosci.

[199] Cox K, Combs B, Abdelmesih B, Morfini G, Brady ST, Kanaan NM (2016). Analysis of isoform-specific tau aggregates suggests a common toxic mechanism involving similar pathological conformations and axonal transport inhibition. Neurobiol Aging.

[200] Rodriguez-Martin T, Cuchillo-Ibanez I, Noble W, Nyenya F, Anderton BH, Hanger DP (2013). Tau phosphorylation affects its axonal transport and degradation. Neurobiol Aging.

[201] Maziuk BF, Apicco DJ, Cruz AL, Jiang L, Ash PEA, da Rocha EL, Zhang C, Yu WH, Leszyk J, Abisambra JF, Li H, Wolozin B (2018). RNA binding proteins co-localize with small tau inclusions in tauopathy. Acta Neuropathol Commun.

[202] Apicco DJ, Ash PEA, Maziuk B, LeBlang C, Medalla M, Al Abdullatif A, Ferragud A, Botelho E, Ballance HI, Dhawan U, Boudeau S, Cruz AL, Kashy D, Wong A, Goldberg LR, Yazdani N, Zhang C, Ung CY, Tripodis Y, Kanaan NM, Ikezu T, Cottone P, Leszyk J, Li H, Luebke J, Bryant CD, Wolozin B (2018). Reducing the RNA binding protein TIA1 protects against tau-mediated neurodegeneration in vivo. Nat Neurosci.

[203] Yoshitake J, Soeda Y, Ida T, Sumioka A, Yoshikawa M, Matsushita K, Akaike T, Takashima A (2016). Modification of TAU by 8-Nitroguanosine 3’,5’-cyclic monophosphate (8-nitro-cGMP): EFFECTS OF NITRIC OXIDE-LINKED CHEMICAL MODIFICATION ON TAU AGGREGATION. J Biol Chem.

[204] Brunello CA, Yan X, Huttunen HJ (2016). Internalized tau sensitizes cells to stress by promoting formation and stability of stress granules. Sci Rep.

[205] Pallas-Bazarra N, Jurado-Arjona J, Navarrete M, Esteban JA, Hernandez F, Avila J, Llorens-Martin M (2016). Novel function of tau in regulating the effects of external stimuli on adult hippocampal neurogenesis. EMBO J.

[206] Vanderweyde T, Apicco DJ, Youmans-Kidder K, Ash PEA, Cook C, Lummertz da Rocha E, Jansen-West K, Frame AA, Citro A, Leszyk JD, Ivanov P, Abisambra JF, Steffen M, Li H, Petrucelli L, Wolozin B (2016). Interaction of tau with the RNA-binding protein TIA1 regulates tau pathophysiology and toxicity. Cell Rep.

[207] Kohler C (2016). Granulovacuolar degeneration: a neurodegenerative change that accompanies tau pathology. Acta Neuropathol.

[208] Wren MC, Zhao J, Liu CC, Murray ME, Atagi Y, Davis MD, Fu Y, Okano HJ, Ogaki K, Strongosky AJ, Tacik P, Rademakers R, Ross OA, Dickson DW, Wszolek ZK, Kanekiyo T, Bu G (2015). Frontotemporal dementia-associated N279K tau mutant disrupts subcellular vesicle trafficking and induces cellular stress in iPSC-derived neural stem cells. Mol Neurodegener.

[209] Komuro Y, Xu G, Bhaskar K, Lamb BT (2015). Human tau expression reduces adult neurogenesis in a mouse model of tauopathy. Neurobiol Aging.

[210] Joseph M, Anglada-Huguet M, Paesler K, Mandelkow E, Mandelkow EM (2017). Anti-aggregant tau mutant promotes neurogenesis. Mol Neurodegener.

[211] Dioli C, Patricio P, Trindade R, Pinto LG, Silva JM, Morais M, Ferreiro E, Borges S, Mateus-Pinheiro A, Rodrigues AJ, Sousa N, Bessa JM, Pinto L, Sotiropoulos I (2017). Tau-dependent suppression of adult neurogenesis in the stressed hippocampus. Mol Psychiatry.

[212] Colonna M, Butovsky O (2017). Microglia function in the central nervous system during health and neurodegeneration. Annu Rev Immunol.

[213] Woodling NS, Andreasson KI (2016). Untangling the web: toxic and protective effects of Neuroinflammation and PGE2 signaling in Alzheimer’s Disease. ACS Chem Neurosci.

[214] Labzin LI, Heneka MT, Latz E (2018). Innate immunity and neurodegeneration. Annu Rev Med.

[215] Mushegian AA. Harnessing innate immunity to fight neurodegeneration. Sci Signal. 2017;10. 10.1126/scisignal.aam8102 28119462

[216] Bolos M, Llorens-Martin M, Perea JR, Jurado-Arjona J, Rabano A, Hernandez F, Avila J (2017). Absence of CX3CR1 impairs the internalization of tau by microglia. Mol Neurodegener.

[217] Laurent C, Dorothee G, Hunot S, Martin E, Monnet Y, Duchamp M, Dong Y, Legeron FP, Leboucher A, Burnouf S, Faivre E, Carvalho K, Caillierez R, Zommer N, Demeyer D, Jouy N, Sazdovitch V, Schraen-Maschke S, Delarasse C, Buee L, Blum D (2017). Hippocampal T cell infiltration promotes neuroinflammation and cognitive decline in a mouse model of tauopathy. Brain.

[218] Bellucci A, Westwood AJ, Ingram E, Casamenti F, Goedert M, Spillantini MG (2004). Induction of inflammatory mediators and microglial activation in mice transgenic for mutant human P301S tau protein. Am J Pathol.

[219] Leyns CEG, Holtzman DM (2017). Glial contributions to neurodegeneration in tauopathies. Mol Neurodegener.

[220] Frost B, Jacks RL, Diamond MI (2009). Propagation of tau misfolding from the outside to the inside of a cell. J Biol Chem.

[221] Lewis J, Dickson DW (2016). Propagation of tau pathology: hypotheses, discoveries, and yet unresolved questions from experimental and human brain studies. Acta Neuropathol.

[222] Wang Y, Balaji V, Kaniyappan S, Kruger L, Irsen S, Tepper K, Chandupatla R, Maetzler W, Schneider A, Mandelkow E, Mandelkow EM (2017). The release and trans-synaptic transmission of tau via exosomes. Mol Neurodegener.

[223] Goedert M, Eisenberg DS, Crowther RA (2017). Propagation of tau aggregates and neurodegeneration. Annu Rev Neurosci.

[224] Polanco JC, Scicluna BJ, Hill AF, Gotz J (2016). Extracellular vesicles isolated from the brains of rTg4510 mice seed tau protein aggregation in a threshold-dependent manner. J Biol Chem.

[225] Boluda S, Iba M, Zhang B, Raible KM, Lee VM, Trojanowski JQ (2015). Differential induction and spread of tau pathology in young PS19 tau transgenic mice following intracerebral injections of pathological tau from Alzheimer’s disease or corticobasal degeneration brains. Acta Neuropathol.

[226] Xu Y, Martini-Stoica H, Zheng H (2016). A seeding based cellular assay of tauopathy. Mol Neurodegener.

[227] Stanley M, Macauley SL, Holtzman DM (2016). Changes in insulin and insulin signaling in Alzheimer’s disease: cause or consequence?. J Exp Med.

[228] Biessels GJ, Reagan LP (2015). Hippocampal insulin resistance and cognitive dysfunction. Nat Rev Neurosci.

[229] Blazquez E, Velazquez E, Hurtado-Carneiro V, Ruiz-Albusac JM (2014). Insulin in the brain: its pathophysiological implications for states related with central insulin resistance, type 2 diabetes and Alzheimer’s disease. Front Endocrinol (Lausanne).

[230] Talbot K, Wang HY, Kazi H, Han LY, Bakshi KP, Stucky A, Fuino RL, Kawaguchi KR, Samoyedny AJ, Wilson RS, Arvanitakis Z, Schneider JA, Wolf BA, Bennett DA, Trojanowski JQ, Arnold SE (2012). Demonstrated brain insulin resistance in Alzheimer’s disease patients is associated with IGF-1 resistance, IRS-1 dysregulation, and cognitive decline. J Clin Invest.

[231] Rodriguez-Rodriguez P, Sandebring-Matton A, Merino-Serrais P, Parrado-Fernandez C, Rabano A, Winblad B, Avila J, Ferrer I, Cedazo-Minguez A (2017). Tau hyperphosphorylation induces oligomeric insulin accumulation and insulin resistance in neurons. Brain.

[232] Gratuze M, Joly-Amado A, Vieau D, Buee L, Blum D (2018). Mutual relationship between tau and central insulin Signalling: consequences for AD and Tauopathies?. Neuroendocrinology.

[233] Barini E, Antico O, Zhao Y, Asta F, Tucci V, Catelani T, Marotta R, Xu H, Gasparini L (2016). Metformin promotes tau aggregation and exacerbates abnormal behavior in a mouse model of tauopathy. Mol Neurodegener.

[234] Holtzman DM, Carrillo MC, Hendrix JA, Bain LJ, Catafau AM, Gault LM, Goedert M, Mandelkow E, Mandelkow EM, Miller DS, Ostrowitzki S, Polydoro M, Smith S, Wittmann M, Hutton M (2016). Tau: from research to clinical development. Alzheimers Dement.

[235] Spillantini MG, Murrell JR, Goedert M, Farlow MR, Klug A, Ghetti B (1998). Mutation in the tau gene in familial multiple system tauopathy with presenile dementia. Proc Natl Acad Sci U S A.

[236] Hutton M, Lendon CL, Rizzu P, Baker M, Froelich S, Houlden H, Pickering-Brown S, Chakraverty S, Isaacs A, Grover A, Hackett J, Adamson J, Lincoln S, Dickson D, Davies P, Petersen RC, Stevens M, de Graaff E, Wauters E, van Baren J, Hillebrand M, Joosse M, Kwon JM, Nowotny P, Che LK, Norton J, Morris JC, Reed LA, Trojanowski J, Basun H, Lannfelt L, Neystat M, Fahn S, Dark F, Tannenberg T, Dodd PR, Hayward N, Kwok JB, Schofield PR, Andreadis A, Snowden J, Craufurd D, Neary D, Owen F, Oostra BA, Hardy J, Goate A, van Swieten J, Mann D, Lynch T, Heutink P (1998). Association of missense and 5′-splice-site mutations in tau with the inherited dementia FTDP-17. Nature.

[237] Pir GJ, Choudhary B, Mandelkow E, Mandelkow EM (2016). Tau mutant A152T, a risk factor for FTD/PSP, induces neuronal dysfunction and reduced lifespan independently of aggregation in a C. elegans Tauopathy model. Mol Neurodegener.

[238] Braak H, Del Tredici K (2011). The pathological process underlying Alzheimer’s disease in individuals under thirty. Acta Neuropathol.

[239] Braak H, Thal DR, Ghebremedhin E, Del Tredici K (2011). Stages of the pathologic process in Alzheimer disease: age categories from 1 to 100 years. J Neuropathol Exp Neurol.

[240] Zhao N, Liu CC, Van Ingelgom AJ, Linares C, Kurti A, Knight JA, Heckman MG, Diehl NN, Shinohara M, Martens YA, Attrebi ON, Petrucelli L, Fryer JD, Wszolek ZK, Graff-Radford NR, Caselli RJ, Sanchez-Contreras MY, Rademakers R, Murray ME, Koga S, Dickson DW, Ross OA, Bu G (2018). APOE epsilon2 is associated with increased tau pathology in primary tauopathy. Nat Commun.

[241] Ikeda K, Akiyama H, Arai T, Sahara N, Mori H, Usami M, Sakata M, Mizutani T, Wakabayashi K, Takahashi H (1997). A subset of senile dementia with high incidence of the apolipoprotein E epsilon2 allele. Ann Neurol.

[242] Ghebremedhin E, Schultz C, Botez G, Rub U, Sassin I, Braak E, Braak H (1998). Argyrophilic grain disease is associated with apolipoprotein E epsilon 2 allele. Acta Neuropathol.

[243] Vos SJ, Xiong C, Visser PJ, Jasielec MS, Hassenstab J, Grant EA, Cairns NJ, Morris JC, Holtzman DM, Fagan AM (2013). Preclinical Alzheimer’s disease and its outcome: a longitudinal cohort study. Lancet Neurol.

[244] Mattsson N, Zetterberg H, Hansson O, Andreasen N, Parnetti L, Jonsson M, Herukka SK, van der Flier WM, Blankenstein MA, Ewers M, Rich K, Kaiser E, Verbeek M, Tsolaki M, Mulugeta E, Rosen E, Aarsland D, Visser PJ, Schroder J, Marcusson J, de Leon M, Hampel H, Scheltens P, Pirttila T, Wallin A, Jonhagen ME, Minthon L, Winblad B, Blennow K (2009). CSF biomarkers and incipient Alzheimer disease in patients with mild cognitive impairment. JAMA.

[245] Hansson O, Zetterberg H, Buchhave P, Londos E, Blennow K, Minthon L (2006). Association between CSF biomarkers and incipient Alzheimer’s disease in patients with mild cognitive impairment: a follow-up study. Lancet Neurol.

[246] Gauthier S, Feldman HH, Schneider LS, Wilcock GK, Frisoni GB, Hardlund JH, Moebius HJ, Bentham P, Kook KA, Wischik DJ, Schelter BO, Davis CS, Staff RT, Bracoud L, Shamsi K, Storey JM, Harrington CR, Wischik CM (2016). Efficacy and safety of tau-aggregation inhibitor therapy in patients with mild or moderate Alzheimer’s disease: a randomised, controlled, double-blind, parallel-arm, phase 3 trial. Lancet.

[247] Novak P, Schmidt R, Kontsekova E, Zilka N, Kovacech B, Skrabana R, Vince-Kazmerova Z, Katina S, Fialova L, Prcina M, Parrak V, Dal-Bianco P, Brunner M, Staffen W, Rainer M, Ondrus M, Ropele S, Smisek M, Sivak R, Winblad B, Novak M (2017). Safety and immunogenicity of the tau vaccine AADvac1 in patients with Alzheimer’s disease: a randomised, double-blind, placebo-controlled, phase 1 trial. Lancet Neurol.

[248] Medina M. An Overview on the Clinical Development of Tau-Based Therapeutics. Int J Mol Sci. 2018;19. 10.3390/ijms19041160 PMC5979300 29641484

[249] Agadjanyan MG, Zagorski K, Petrushina I, Davtyan H, Kazarian K, Antonenko M, Davis J, Bon C, Blurton-Jones M, Cribbs DH, Ghochikyan A (2017). Humanized monoclonal antibody armanezumab specific to N-terminus of pathological tau: characterization and therapeutic potency. Mol Neurodegener.

[250] Congdon EE, Lin Y, Rajamohamedsait HB, Shamir DB, Krishnaswamy S, Rajamohamedsait WJ, Rasool S, Gonzalez V, Levenga J, Gu J, Hoeffer C, Sigurdsson EM (2016). Affinity of tau antibodies for solubilized pathological tau species but not their immunogen or insoluble tau aggregates predicts in vivo and ex vivo efficacy. Mol Neurodegener.

[251] Ising C, Gallardo G, Leyns CEG, Wong CH, Stewart F, Koscal LJ, Roh J, Robinson GO, Remolina Serrano J, Holtzman DM (2017). AAV-mediated expression of anti-tau scFvs decreases tau accumulation in a mouse model of tauopathy. J Exp Med.

[252] West T, Hu Y, Verghese PB, Bateman RJ, Braunstein JB, Fogelman I, Budur K, Florian H, Mendonca N, Holtzman DM (2017). Preclinical and clinical development of ABBV-8E12, a humanized anti-tau antibody, for treatment of Alzheimer’s Disease and other Tauopathies. J Prev Alzheimers Dis.

[253] Yanamandra K, Patel TK, Jiang H, Schindler S, Ulrich JD, Boxer AL, Miller BL, Kerwin DR, Gallardo G, Stewart F, Finn MB, Cairns NJ, Verghese PB, Fogelman I, West T, Braunstein J, Robinson G, Keyser J, Roh J, Knapik SS, Hu Y, Holtzman DM. Anti-tau antibody administration increases plasma tau in transgenic mice and patients with tauopathy. Sci Transl Med. 2017;9. 10.1126/scitranslmed.aal2029 PMC5727571 28424326

[254] Strang KH, Goodwin MS, Riffe C, Moore BD, Chakrabarty P, Levites Y, Golde TE, Giasson BI (2017). Generation and characterization of new monoclonal antibodies targeting the PHF1 and AT8 epitopes on human tau. Acta Neuropathol Commun.

[255] Jack CR, Wiste HJ, Weigand SD, Therneau TM, Knopman DS, Lowe V, Vemuri P, Mielke MM, Roberts RO, Machulda MM, Senjem ML, Gunter JL, Rocca WA, Petersen RC (2017). Age-specific and sex-specific prevalence of cerebral beta-amyloidosis, tauopathy, and neurodegeneration in cognitively unimpaired individuals aged 50-95 years: a cross-sectional study. Lancet Neurol.

[256] Jack CR, Bennett DA, Blennow K, Carrillo MC, Feldman HH, Frisoni GB, Hampel H, Jagust WJ, Johnson KA, Knopman DS, Petersen RC, Scheltens P, Sperling RA, Dubois B (2016). A/T/N: an unbiased descriptive classification scheme for Alzheimer disease biomarkers. Neurology.

[257] Fulop T, Itzhaki RF, Balin BJ, Miklossy J, Barron AE (2018). Role of microbes in the development of Alzheimer’s Disease: state of the art - an international symposium presented at the 2017 IAGG congress in San Francisco. Front Genet.

[258] Doulberis M, Kotronis G, Thomann R, Polyzos SA, Boziki M, Gialamprinou D, Deretzi G, Katsinelos P, Kountouras J. Review: impact of helicobacter pylori on Alzheimer’s disease: what do we know so far? Helicobacter. 2018;23. 10.1111/hel.12454 29181894

[259] Harris SA, Harris EA (2018). Molecular mechanisms for herpes simplex virus type 1 pathogenesis in Alzheimer’s Disease. Front Aging Neurosci.

[260] Itzhaki RF, Lathe R, Balin BJ, Ball MJ, Bearer EL, Braak H, Bullido MJ, Carter C, Clerici M, Cosby SL, Del Tredici K, Field H, Fulop T, Grassi C, Griffin WS, Haas J, Hudson AP, Kamer AR, Kell DB, Licastro F, Letenneur L, Lovheim H, Mancuso R, Miklossy J, Otth C, Palamara AT, Perry G, Preston C, Pretorius E, Strandberg T, Tabet N, Taylor-Robinson SD, Whittum-Hudson JA (2016). Microbes and Alzheimer’s Disease. J Alzheimers Dis.

[261] Harding A, Gonder U, Robinson SJ, Crean S, Singhrao SK (2017). Exploring the association between Alzheimer’s Disease, Oral Health, Microbial Endocrinology and Nutrition. Front Aging Neurosci.

[262] Kristensson K, Masocha W, Bentivoglio M (2013). Mechanisms of CNS invasion and damage by parasites. Handb Clin Neurol.

[263] Dando SJ, Mackay-Sim A, Norton R, Currie BJ, St John JA, Ekberg JA, Batzloff M, Ulett GC, Beacham IR (2014). Pathogens penetrating the central nervous system: infection pathways and the cellular and molecular mechanisms of invasion. Clin Microbiol Rev.

[264] Dahm T, Rudolph H, Schwerk C, Schroten H, Tenenbaum T (2016). Neuroinvasion and inflammation in viral central nervous system infections. Mediat Inflamm.

[265] Doran KS, Fulde M, Gratz N, Kim BJ, Nau R, Prasadarao N, Schubert-Unkmeir A, Tuomanen EI, Valentin-Weigand P (2016). Host-pathogen interactions in bacterial meningitis. Acta Neuropathol.

[266] Miner JJ, Diamond MS (2016). Mechanisms of restriction of viral neuroinvasion at the blood-brain barrier. Curr Opin Immunol.

[267] Solomos AC, Rall GF (2016). Get it through your thick Head: emerging principles in Neuroimmunology and Neurovirology redefine central nervous system “immune privilege”. ACS Chem Neurosci.

[268] Ball MJ (1982). Limbic predilection in Alzheimer dementia: is reactivated herpesvirus involved?. Can J Neurol Sci.

[269] Itzhaki RF, Wozniak MA, Appelt DM, Balin BJ (2004). Infiltration of the brain by pathogens causes Alzheimer’s disease. Neurobiol Aging.

[270] Itzhaki RF (2014). Herpes simplex virus type 1 and Alzheimer’s disease: increasing evidence for a major role of the virus. Front Aging Neurosci.

[271] Itzhaki RF (2016). Herpes and Alzheimer’s Disease: subversion in the central nervous system and how it might be halted. J Alzheimers Dis.

[272] Itzhaki RF (2017). Herpes simplex virus type 1 and Alzheimer’s disease: possible mechanisms and signposts. FASEB J.

[273] Singhrao SK, Harding A, Simmons T, Robinson S, Kesavalu L, Crean S (2014). Oral inflammation, tooth loss, risk factors, and association with progression of Alzheimer’s disease. J Alzheimers Dis.

[274] Gurav A (1992). N. (2014) Alzheimer’s disease and periodontitis--an elusive link. Rev Assoc Med Bras.

[275] Pritchard AB, Crean S, Olsen I, Singhrao SK (2017). Periodontitis, Microbiomes and their Role in Alzheimer’s Disease. Front Aging Neurosci.

[276] Aguayo S, Schuh C, Vicente B, Aguayo LG (2018). Association between Alzheimer’s Disease and Oral and gut microbiota: are pore forming proteins the missing link?. J Alzheimers Dis.

[277] Fulop T, Witkowski JM, Bourgade K, Khalil A, Zerif E, Larbi A, Hirokawa K, Pawelec G, Bocti C, Lacombe G, Dupuis G, Frost EH (2018). Can an infection hypothesis explain the Beta amyloid hypothesis of Alzheimer’s Disease?. Front Aging Neurosci.

[278] Broxmeyer L. Dr. Oskar Fischer’s mysterious little Alzheimer’s germ. J Alzheimers Dis Ed Blog. 2017.

[279] Goedert M (2009). Oskar Fischer and the study of dementia. Brain.

[280] Buxbaum JN (2017). Alzheimer’s Disease: It’s more than Abeta. FASEB J.

[281] Tzeng NS, Chung CH, Lin FH, Chiang CP, Yeh CB, Huang SY, Lu RB, Chang HA, Kao YC, Yeh HW, Chiang WS, Chou YC, Tsao CH, Wu YF, Chien WC (2018). Anti-herpetic medications and reduced risk of dementia in patients with herpes simplex virus infections-a Nationwide, Population-Based Cohort Study in Taiwan. Neurotherapeutics.

[282] Readhead B, Haure-Mirande JV, Funk CC, Richards MA, Shannon P, Haroutunian V, Sano M, Liang WS, Beckmann ND, Price ND, Reiman EM, Schadt EE, Ehrlich ME, Gandy S, Dudley JT (2018). Multiscale analysis of independent Alzheimer’s cohorts finds disruption of molecular, genetic, and clinical networks by human herpesvirus. Neuron.

[283] Chen CK, Wu YT, Chang YC (2017). Association between chronic periodontitis and the risk of Alzheimer’s disease: a retrospective, population-based, matched-cohort study. Alzheimers Res Ther.

[284] Jamieson GA, Maitland NJ, Wilcock GK, Craske J, Itzhaki RF (1991). Latent herpes simplex virus type 1 in normal and Alzheimer’s disease brains. J Med Virol.

[285] Jamieson GA, Maitland NJ, Craske J, Wilcock GK, Itzhaki RF (1991). Detection of herpes simplex virus type 1 DNA sequences in normal and Alzheimer’s disease brain using polymerase chain reaction. Biochem Soc Trans.

[286] Jamieson GA, Maitland NJ, Itzhaki RF (1992). Herpes simplex virus type 1 DNA sequences are present in aged normal and Alzheimer’s disease brain but absent in lymphocytes. Arch Gerontol Geriatr.

[287] Itzhaki RF, Lin WR (1998). Herpes simplex virus type I in brain and the type 4 allele of the apolipoprotein E gene are a combined risk factor for Alzheimer’s disease. Biochem Soc Trans.

[288] Lin WR, Wozniak MA, Cooper RJ, Wilcock GK, Itzhaki RF (2002). Herpesviruses in brain and Alzheimer’s disease. J Pathol.

[289] Wozniak MA, Shipley SJ, Combrinck M, Wilcock GK, Itzhaki RF (2005). Productive herpes simplex virus in brain of elderly normal subjects and Alzheimer’s disease patients. J Med Virol.

[290] Wozniak MA, Mee AP, Itzhaki RF (2009). Herpes simplex virus type 1 DNA is located within Alzheimer’s disease amyloid plaques. J Pathol.

[291] Liedtke W, Opalka B, Zimmermann CW, Lignitz E (1993). Age distribution of latent herpes simplex virus 1 and varicella-zoster virus genome in human nervous tissue. J Neurol Sci.

[292] Nicoll JA, Love S, Kinrade E (1993). Distribution of herpes simplex virus DNA in the brains of human long-term survivors of encephalitis. Neurosci Lett.

[293] Sanders VJ, Felisan S, Waddell A, Tourtellotte WW (1996). Detection of herpesviridae in postmortem multiple sclerosis brain tissue and controls by polymerase chain reaction. J Neuro-Oncol.

[294] Sanders VJ, Felisan SL, Waddell AE, Conrad AJ, Schmid P, Swartz BE, Kaufman M, Walsh GO, De Salles AA, Tourtellotte WW (1997). Presence of herpes simplex DNA in surgical tissue from human epileptic seizure foci detected by polymerase chain reaction: preliminary study. Arch Neurol.

[295] Beffert U, Bertrand P, Champagne D, Gauthier S, Poirier J (1998). HSV-1 in brain and risk of Alzheimer’s disease. Lancet.

[296] Hemling N, Roytta M, Rinne J, Pollanen P, Broberg E, Tapio V, Vahlberg T, Hukkanen V (2003). Herpesviruses in brains in Alzheimer’s and Parkinson’s diseases. Ann Neurol.

[297] Agostini S, Mancuso R, Baglio F, Cabinio M, Hernis A, Guerini FR, Calabrese E, Nemni R, Clerici M (2016). Lack of evidence for a role of HHV-6 in the pathogenesis of Alzheimer’s disease. J Alzheimers Dis.

[298] Hill JM, Ball MJ, Neumann DM, Azcuy AM, Bhattacharjee PS, Bouhanik S, Clement C, Lukiw WJ, Foster TP, Kumar M, Kaufman HE, Thompson HW (2008). The high prevalence of herpes simplex virus type 1 DNA in human trigeminal ganglia is not a function of age or gender. J Virol.

[299] Yamazaki Y, Painter MM, Bu G, Kanekiyo T (2016). Apolipoprotein E as a therapeutic target in Alzheimer’s Disease: A review of basic research and clinical evidence. CNS Drugs.

[300] Itzhaki RF, Lin WR, Shang D, Wilcock GK, Faragher B, Jamieson GA (1997). Herpes simplex virus type 1 in brain and risk of Alzheimer’s disease. Lancet.

[301] Vogt NM, Kerby RL, Dill-McFarland KA, Harding SJ, Merluzzi AP, Johnson SC, Carlsson CM, Asthana S, Zetterberg H, Blennow K, Bendlin BB, Rey FE (2017). Gut microbiome alterations in Alzheimer’s disease. Sci Rep.

[302] Xu R, Wang Q (2016). Towards understanding brain-gut-microbiome connections in Alzheimer’s disease. BMC Syst Biol.

[303] MahmoudianDehkordi S, Arnold M, Nho K, Ahmad S, Jia W, Xie G, Louie G, Kueider-Paisley A, Moseley MA, Thompson JW, St John Williams L, Tenenbaum JD, Blach C, Baillie R, Han X, Bhattacharyya S, Toledo JB, Schafferer S, Klein S, Koal T, Risacher SL, Kling MA, Motsinger-Reif A, Rotroff DM, Jack J, Hankemeier T, Bennett DA, De Jager PL, Trojanowski JQ, Shaw LM, Weiner MW, Doraiswamy PM, van Duijn CM, Saykin AJ, Kastenmuller G, Kaddurah-Daouk R, Alzheimer’s Disease Neuroimaging, I., and the Alzheimer Disease Metabolomics, C. Altered bile acid profile associates with cognitive impairment in Alzheimer’s disease-an emerging role for gut microbiome. Alzheimers Dement. 2018. 10.1016/j.jalz.2018.07.217 PMC6487485 30337151

[304] Kohler CA, Maes M, Slyepchenko A, Berk M, Solmi M, Lanctot KL, Carvalho AF (2016). The gut-brain Axis, Including the Microbiome, Leaky Gut and Bacterial Translocation: Mechanisms and Pathophysiological Role in Alzheimer’s Disease. Curr Pharm Des.

[305] Sochocka M, Donskow-Lysoniewska K, Diniz BS, Kurpas D, Brzozowska E, Leszek J. The gut microbiome alterations and inflammation-driven pathogenesis of Alzheimer’s Disease-a critical review. Mol Neurobiol. 2018. 10.1007/s12035-018-1188-4 PMC6394610 29936690

[306] Minter MR, Hinterleitner R, Meisel M, Zhang C, Leone V, Zhang X, Oyler-Castrillo P, Zhang X, Musch MW, Shen X, Jabri B, Chang EB, Tanzi RE, Sisodia SS (2017). Antibiotic-induced perturbations in microbial diversity during post-natal development alters amyloid pathology in an aged APPSWE/PS1DeltaE9 murine model of Alzheimer’s disease. Sci Rep.

[307] Minter MR, Zhang C, Leone V, Ringus DL, Zhang X, Oyler-Castrillo P, Musch MW, Liao F, Ward JF, Holtzman DM, Chang EB, Tanzi RE, Sisodia SS (2016). Antibiotic-induced perturbations in gut microbial diversity influences neuro-inflammation and amyloidosis in a murine model of Alzheimer’s disease. Sci Rep.

[308] Harach T, Marungruang N, Duthilleul N, Cheatham V, Mc Coy KD, Frisoni G, Neher JJ, Fak F, Jucker M, Lasser T, Bolmont T (2017). Reduction of Abeta amyloid pathology in APPPS1 transgenic mice in the absence of gut microbiota. Sci Rep.

[309] Ball MJ, Lukiw WJ, Kammerman EM, Hill JM (2013). Intracerebral propagation of Alzheimer’s disease: strengthening evidence of a herpes simplex virus etiology. Alzheimers Dement.

[310] Soscia SJ, Kirby JE, Washicosky KJ, Tucker SM, Ingelsson M, Hyman B, Burton MA, Goldstein LE, Duong S, Tanzi RE, Moir RD (2010). The Alzheimer’s disease-associated amyloid beta-protein is an antimicrobial peptide. PLoS One.

[311] Kumar DK, Choi SH, Washicosky KJ, Eimer WA, Tucker S, Ghofrani J, Lefkowitz A, McColl G, Goldstein LE, Tanzi RE, Moir RD (2016). Amyloid-beta peptide protects against microbial infection in mouse and worm models of Alzheimer’s disease. Sci Transl Med.

[312] Eimer WA, Vijaya Kumar DK, Navalpur Shanmugam NK, Rodriguez AS, Mitchell T, Washicosky KJ, Gyorgy B, Breakefield XO, Tanzi RE, Moir RD (2018). Alzheimer’s Disease-associated beta-amyloid is rapidly seeded by Herpesviridae to protect against brain infection. Neuron.

[313] White MR, Kandel R, Tripathi S, Condon D, Qi L, Taubenberger J, Hartshorn KL (2014). Alzheimer’s associated beta-amyloid protein inhibits influenza A virus and modulates viral interactions with phagocytes. PLoS One.

[314] Bourgade K, Garneau H, Giroux G, Le Page AY, Bocti C, Dupuis G, Frost EH, Fulop T (2015). Beta-amyloid peptides display protective activity against the human Alzheimer’s disease-associated herpes simplex virus-1. Biogerontology.

[315] Moir RD, Lathe R, Tanzi RE (2018). The antimicrobial protection hypothesis of Alzheimer’s disease. Alzheimers Dement.

[316] Lee MH, Siddoway B, Kaeser GE, Segota I, Rivera R, Romanow WJ, Liu CS, Park C, Kennedy G, Long T, Chun J. Somatic APP gene recombination in Alzheimer’s disease and normal neurons. Nature. 2018. 10.1038/s41586-018-0718-6 PMC6391999 30464338

